# Selective Schiff Base Formation of Group 9 Organometallic Complexes with Functionalized Spirobifluorene Ligands

**DOI:** 10.3390/molecules28207155

**Published:** 2023-10-18

**Authors:** Krystal M. Cid-Seara, Raquel Pereira-Cameselle, Sandra Bolaño, Maria Talavera

**Affiliations:** 1 Departamento de Química Inorgánica, Universidade de Vigo, Campus Universitario, 36310 Vigo, Spain; 2 Departamento de Química Orgánica, Universidade de Vigo, Campus Universitario, 36310 Vigo, Spain

**Keywords:** iridium, rhodium, spirobifluorene, amines

## Abstract

Organic amines are important compounds present in a wide variety of products, which makes the development of new systems for their detection an interesting field of study. New organometallic complexes of group 9 [MCp*X(2′-R-2-py-SBF)] (M = Ir, Rh; R = H, X = Cl (**6**), R = H, X = OAc (**7**), R = CHO, X = Cl (**8**)), and [IrCp*Cl(2′, 7-diCHO-2-py-SBF)] (**9**) (Cp* pentamethylcyclopentadienyl, SBF = 9,9’-spirobifluorene) bearing bidentate C–N ligands based on 9,9′-spirobifluorene were obtained and characterized by NMR spectroscopy, mass spectrometry, IR spectroscopy, and X-ray diffraction analysis when possible. The formation of a Schiff base to give complexes with the formula [MCp*Cl(2′-CH=NR-2-py-SBF)] (M = Ir, Rh; R = alkyl or aryl (**10**–**12**)), through condensation of an amine, and the aldehyde group present in these new complexes was studied leading to a selective reactivity depending on the nature of the amine and the metal center. While the iridium complexes only react with aromatic amines, the rhodium derivative requires heat for those but can react at room temperature with aliphatic amines.

## 1. Introduction

Organometallic complexes as sensors in the detection of amines are of vital importance due to the presence of these organic derivatives in pharmaceutical, organic, and food products, as well as in our organisms, where they fundamentally control many of the physiological functions [1]. For example, the coordination of amines to iridium compounds has been previously described for generating luminescence, which makes them good candidates as sensors [2,3]. Another way of detecting amines is through the formation of imine derivatives, Schiff bases [4,5,6,7]. Thus, chiral aldehydes have been used with chiral amine compounds, giving rise to diastereomeric imines, where the ^1^H NMR spectroscopy technique allows the enantiomeric purity of the amines to be determined [5]. 

**Figure 1:**
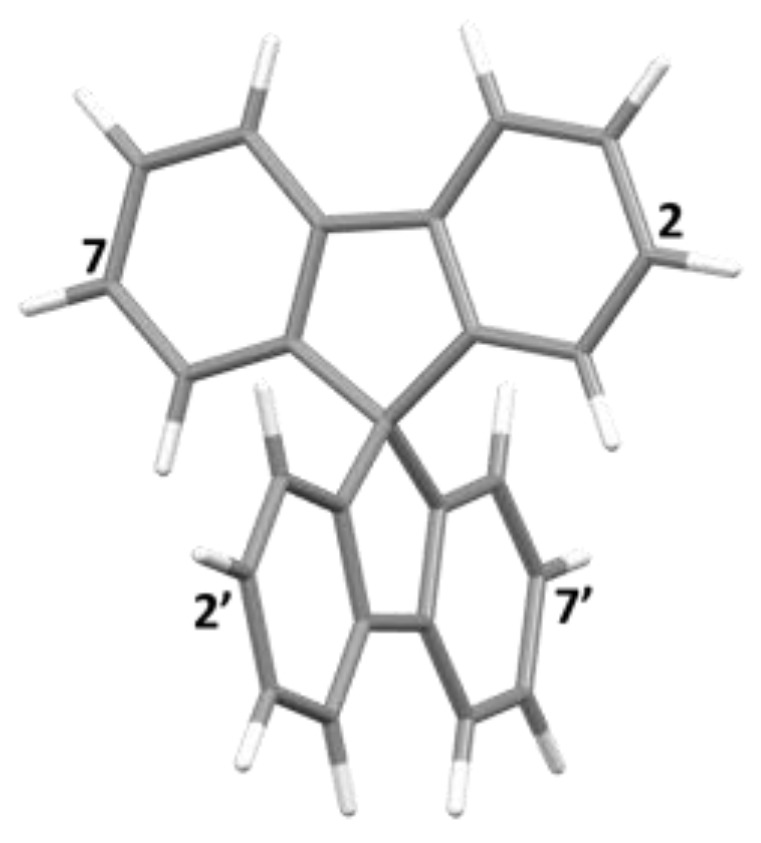
Representation of 9,9′-spirobifluorene labeling the substitution positions.

Cyclometallated complexes with bidentate C–N ligands are well-known and have been used with good results as amine detectors [2,5,8,9]. Among the bidentate C–N ligands, those based on 9,9′-spirobifluorene (SBF) appear as interesting ligands as the orthogonal arrangement between the two π-systems of the SBF provides rigidity, a high steric effect, thermal and structural stability [10], and increased solubility in a wide range of organic solvents [11]. In addition, it allows the possibility of inducing axial chirality by introducing substituents at specific positions (2,2’,7,7’) (Figure 1), tuning their properties and extending its field of application to not only chiroptical materials but several other applications [12,13,14,15]. While functionalization of SBF has been mainly developed for chiroptical devices [15,16,17,18,19] such as OLEDs, 2,2′,7,7′-tetrakis-(*N,N*-di-*p*-methoxyphenylamine)-9,9′-spirobifluorene is used as hole transport material in perovskite solar cells [14]. Regarding sensors, the development of imidazolium polymer based on 2,2′,7,7′-tetraimidazole spirobifluorene can easily detect iron (III) or dichromate anion [20]. In addition, 2,2′ substituted SBF-based dendrimers have been used for the detection of explosives [21].

Herein, the synthesis of cyclometallated iridium and rhodium complexes with a bidentate C–N ligand based on 9,9′-spirobifluorene where one of its branches bears an aldehyde substituent is proposed. The group will allow us to analyze the behavior of this type of organometallic complexes towards aliphatic and aromatic amines with a view to their future application as amine detection sensors. 

## 2. Results and Discussion

**Scheme 1:**
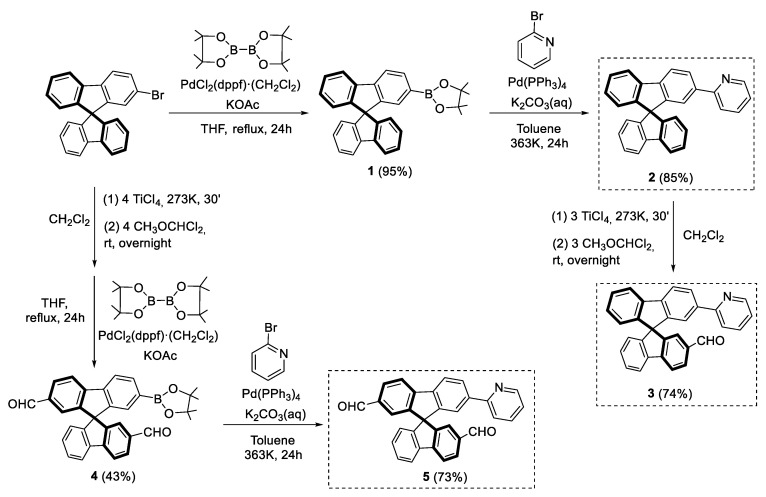
Synthetic routes of the organic compounds used in this work.

The obtaining of the spirobifluorene-functionalized ligands was based on common organic reactions summarized in Scheme 1. Thus, using 2-bromo-9,9′-spirobifluorene as a starting reagent and PdCl_2_(1,1’-bis(diphenylphosphino)ferrocene) as a catalyst, a pinacolboranyl substituent can be introduced to give **1**. This compound reacts through a Suzuki−Miyaura cross-coupling reaction with 2-bromopyridine to provide **2**, a spirobifluorene functionalized with a pyridine ligand. Then, compound **2** reacts with TiCl_4_ and dichloromethyl-methylether as a formyl source by Rieche formylation leading to compound **3**. Thus, compound **3** bears an aldehyde group at the second branch of the spirobifluorene moiety as the presence of a pyridine substituent already in the system produces, apart from a –I effect, a +R effect at position 7 of the same branch promoting the electrophilic aromatic substitution only at the second branch even with a little excess of formylation reagents [22].

In addition, a double-formylated compound could be obtained. To avoid deactivating processes, the first step was the diformylation from the bromo-substituted derivative. In this case, the bromine group only presents an inductive effect (-I); therefore, the absence of the +R effect avoids the hampering of the diformylation in a stoichiometric reaction. Note that the introduction of the first CHO substituent has a very strong deactivating effect in its branch, promoting the second formylation at the bromine branch. Afterward, the borylation reaction using the same methodology as before was performed. Thus, **4** was obtained bearing an aldehyde group at each spirobifluorene branch, in positions 2′ and 7. Again, the C–C coupling reaction finally provided the targeted compound **5** (see experimental section for characterization of all the organic derivatives). 

**Scheme 2:**
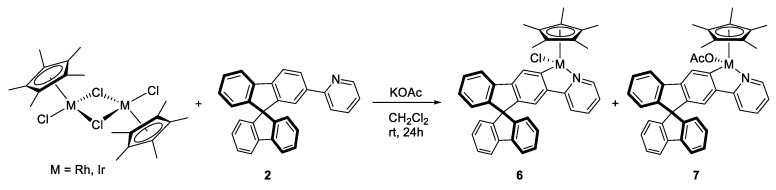
Synthesis of complexes **6** and **7**.

Treatment of compound **2** with the dimeric complex [MCp*(Cl)(*µ*-Cl)]_2_ (M = Ir, Rh) in presence of potassium acetate led to the formation of a mixture of [MCp*Cl(2-py-SBF)] (M = Ir (**Ir6**), Rh (**Rh6**)) and [MCp*(OCOCH_3_)(2-py-SBF)] (M = Ir (**Ir7**), Rh (**Rh7**)) (Cp* = pentamethylcyclopentadienyl, SBF = 9,9‘-spirobifluorene) in an approximate 1:2 ratio for iridium and 1:1.5 ratio for rhodium, respectively(Scheme 2). This ratio was determined by integration at the ^1^H NMR spectra using the Cp* signals as well as the resonance of the CH group directly bonded to the nitrogen atom of the pyridine moiety. Complex **7** stems from a competition between the starting dimers [MCp*(Cl)(*µ*-Cl)]_2_ (M = Ir, Rh) and complex **6** toward a reaction with KOAc. However, lower amounts of acetate or shorter reaction times led to lower conversions of the starting material with the same product ratio, and therefore, salt metathesis could not be avoided. 

For characterization, mass spectrometry was attempted. In the case of the rhodium complexes, the ionization provoked their decomposition, while for iridium, the release of the chloride or acetate ligand was observed with the molecular peak at *m/z* = 720.2218. However, the ligand exchange was suggested by the NMR spectra analysis for both transition metal complexes. Thus, two sets of signals are clearly identified for the iridium complexes in both ^1^H and ^13^C{^1^H} NMR spectra in CD_2_Cl_2_. The acetate ligand is confirmed by the presence of resonance in the ^1^H NMR spectrum at 1.66 ppm integrating by 3H and the carbon resonances in ^13^C{^1^H} NMR spectrum at 177.2 and 23.1 ppm for the carbonyl and the methyl groups, respectively. However, at rhodium complexes, the signals are overlapped in ^1^H NMR where the methyl group of the acetate ligand confirm the presence of complex **Rh7,** and the integration of the signals together with the ^13^C{^1^H} NMR data allows the identification of both compounds. Apart from the acetate ligand of complex **7**, the most characteristic resonances of **6** and **7** are the Cp* signal at around 1.7 ppm and the carbon resonance bonded to the metal (C3). The latter appears at around 164 ppm for iridium as a singlet and 179 ppm as a doublet of 32 Hz for the rhodium complexes due to the one-bond coupling of the carbon atom with the rhodium nucleus. 

**Figure 2:**
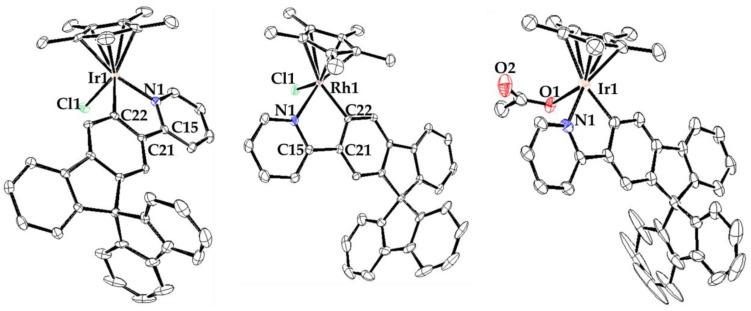
ORTEP representation of complexes **Ir6** (**left**), **Rh6** (**center**), and **Ir7** (**right**) with thermal ellipsoids drawn at a 50% probability level. All hydrogen atoms have been omitted for clarity.

Finally, crystals adequate for X-ray diffraction analysis were obtained for complex **6** (Figure 2, left and center). In addition, low-quality crystals of **Ir7** were also obtained. Although the latter are not suitable for publication, they confirmed the presence of the acetate ligand in the iridium coordination sphere instead of the chloride (Figure 2, right). The asymmetric unit of the metallacycle complexes **Ir6** and **Rh6** only contains the iridium or rhodium neutral complex. In both cases, it consists of the pentamethylcyclopentadienyl ligand *η^5^*-coordinated to the iridium atom and a chloride ligand. The metal atom forms part of a five-member ring due to the coordination of a spirobifluorene *ortho* substituted with a pyridine group in a chelating form to the metal. The five-member ring is almost planar with a deviation of the metal center of 0.099 Å or 0.133 Å for the iridium or the rhodium complex, respectively. Note that the coordination sphere presents a “three-ledge piano stool” structure in an octahedral arrangement. As shown in Table 1, both complexes present very similar bond lengths and angles despite the metal center change. The M–N bond lengths of 2.097(3) Å and 2.094(2) Å are longer than the M–C bond lengths of 2.047(3) Å and 2.027(2) Å with values in the range of those reported in literature for iridium and rhodium mononuclear complexes bearing other bidentate C–N ligands such as 2-phenylpyridinyl (ppy) or 2-(*p*-tolyl)pyridinyl [23,24,25]. Also, the distance between the centroid (CT) of the Cp* ring and the metal center is 1.823 Å and 1.833 Å for iridium and rhodium, respectively. This value is slightly shorter than the 2.104 Å reported for [RhCp*(SPh)(ppy)] [23] but similar to other carbon-based metallacyclic systems [26,27].

**Scheme 3:**
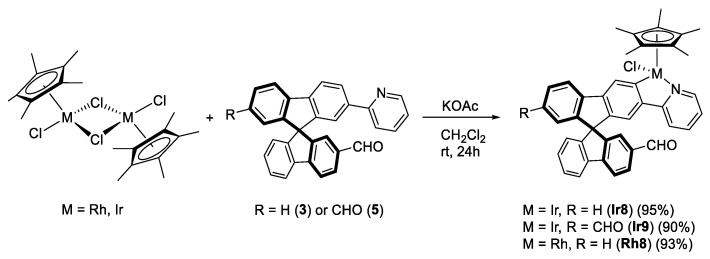
Synthesis of complexes **8** and **Ir9**.

In the search for an organometallic complex bearing an aldehyde group, the formylation reaction of complexes **6** and **7** was attempted without success. Therefore, organic compounds bearing an aldehyde substituent at the spirobifluorene moiety were chosen as ligands. Thus, the reaction of [MCp*(Cl)(*µ*-Cl)]_2_ (M = Ir, Rh) with the spirobifluorene-functionalized compound **3** in the presence of KOAc in CH_2_Cl_2_ led to the synthesis of the new metallacyclic complexes (*R,M*)/(*R,P*)-[MCp*Cl(2′-CHO-2-py-SBF)] (M = Rh (**Rh8**), Ir (**Ir8**)). Similarly, (*R,M*)/(*R,P*)-[IrCp*Cl(2′,7-di(CHO)-2-py-SBF)] (**Ir9**) was also synthesized from **5** (Scheme 3). Note that in these cases, the excess of potassium acetate in the reaction mixture did not produce the ligand exchange, and therefore, complexes bearing an acetate ligand were not observed. In addition, complexes **8** and **Ir9** were obtained as a mixture of diastereomers in around a 60:40 ratio due to the presence of two chiral entities: the iridium atom and the chiral axis at the spirobifluorene moiety as a result of the different substitution at both branches. 

Complexes **8** and **Ir9** were fully characterized by NMR spectroscopy, mass spectrometry, and IR spectroscopy (see experimental for full details). Although the mass spectra show the corresponding isotopic pattern and molecular peak after losing the chloride ligand, the presence of diastereomers can only be determined by the NMR data. Thus, two sets of signals are observed for all the complexes. Particularly, the ^1^H NMR spectra show two signals corresponding to the aldehyde group at 9.75 and 9.88 ppm for **Ir8** and 9.77 and 9.86 ppm for **Rh8**, while complex **Ir9** shows two resonances at 9.87 and 9.82 ppm for one diastereomer and two at 9.83 and 9.73 ppm for the other. Similarly, in ^13^C{^1^H} NMR the aldehydes group are also observed with resonances at 192.2 and 191.9 ppm for **Ir8**, 192.0 and 191.9 ppm for **Rh8** and between 192.0 and 191.8 ppm, the four signals corresponding to **Ir9**. As happened before, the coordination of the spirobifluorene ligand is determined by the disappearance of the C–*H* resonance at position 3, while quaternary carbon atoms are observed at 166.8 and 166.7 ppm for **Ir8** and 166.4 and 166.3 ppm for **Ir9**, all as singlets. For **Rh8**, again, two doublets of 30 Hz due to the coupling to the rhodium atom are observed at 180.0 and 179.9 ppm. Finally, the presence of the aldehyde group is also confirmed by the IR spectra with bands corresponding to the C=O stretching frequency at 1738 and 1691 cm^−1^ for **Ir8** and **Rh8**, respectively, and 1690 and 1600 cm^−1^ for **Ir9**. 

Once these complexes were characterized, their reactivity toward amines was studied. The synthesis of the Schiff bases implies the formation of stoichiometric amounts of water; however, it can be a reversible reaction in the presence of water traces. Therefore, treatment of complex **Ir8** with an excess of amines and in the presence of activated molecular sieves can force the reaction leading to the imine complexes (*R,M*)/(*R,P*)-[MCp*Cl(2‘-CH=NR-2-py-SBF)] (M = Ir, R = Ph (**Ir10**), *o*-NH_2_-C_6_H_4_ (**Ir11**); M = Rh, R = Ph (**Rh10**), CH_2_CH_2_NH_2_ (**Rh12**)) in the reaction mixture. Thus, aniline, *o*-phenylenediamine, and ethylenediamine were used as proof of concept performing the reaction in chloroform in one day, which provided different outcomes (Table 2). The reaction with the aromatic amines (entries 1 and 2) provided, at room temperature, quantitative conversion of complex **Ir8** towards the formation of an imine substituent at the spirobifluorene moiety. However, the aliphatic amine did not react with complex **Ir8** even at high temperatures (entries 3 and 4). Other common reaction conditions for the formation of organic imines, such as heating in ethanol or methanol in the presence of acetic acid as a catalyst (or without it), were also attempted without performing better. 

On the other hand, the rhodium complex **Rh8** was not capable of activating aniline at room temperature and required heating to 343 K to form the imine quantitatively (entries 5 and 6). Interestingly, the metal change to rhodium allowed the reaction of ethylenediamine to form the corresponding imine complex at room temperature (entry 7). 

**Scheme 4:**
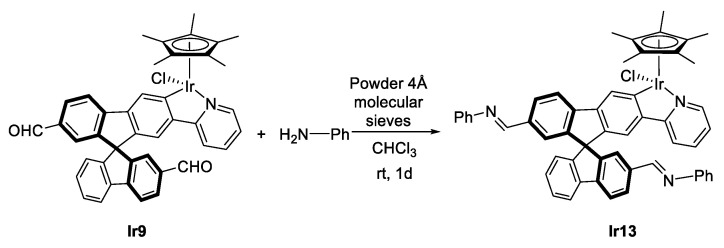
Reactivity of **Ir9** towards aniline.

In order to provide more anchor points for the amine, the complex bearing two aldehyde groups, **Ir9,** reacted toward aniline giving, after one day at room temperature, the diimine complex (*R,M*)/(*R,P*)-[IrCp*Cl(2‘,7-di(CH=NPh)-2-py-SBF)] (**Ir13**) (Scheme 4). As happened with complex **Ir8**, no reaction was observed for ethylenediamine. 

As it has been mentioned, the formation of complexes **Ir10**, **Ir11**, **Ir13**, **Rh10** and **Rh12** can be reversed, leading to instability and difficulties in the full characterization. In some cases, the excess amine was removed successfully, but during characterization, the equilibrium between aldehyde and imine was observed due to possible water traces even on a previously dried solvent. However, in all cases, the ^1^H NMR spectra, together with 2D experiments and the IR spectra, allowed the identification of the characteristic resonances and confirmed their structure (Table 3). Note that, again, the complexes appear as diastereomers due to the stereogenic metal center and the axial chirality provided by the substitution pattern at the spirobifluorene moiety. 

**Figure 3:**
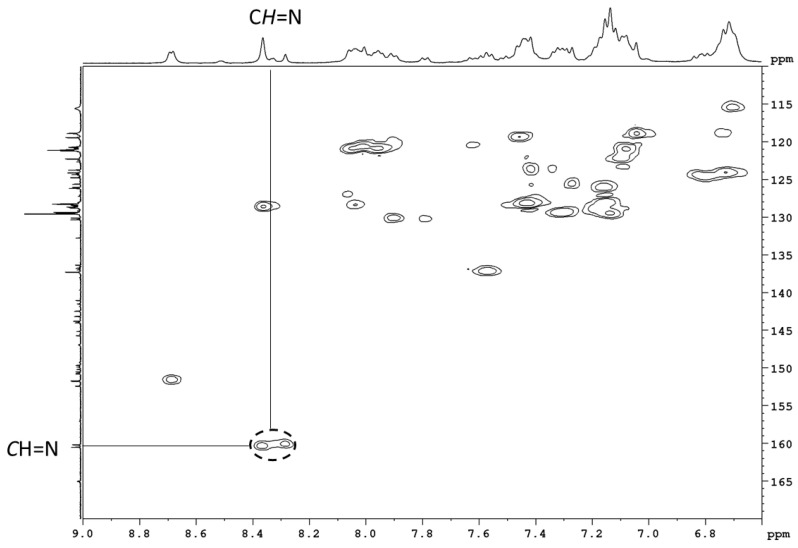
Section of the ^1^H, ^13^C HSQC NMR spectrum of complex **Rh10**.

While the carbon resonances at position 3 in the ^13^C{^1^H} NMR spectrum are similar to the ones described for previous complexes, the CHO resonances at both ^1^H and ^13^C{^1^H} NMR spectra disappear. Instead, new singlets in ^1^H NMR spectra are observed between 8.00 and 8.40 ppm, which correlate in the HSQC NMR experiment with carbon resonances at around 160 ppm, confirming the presence of an imine substituent at the spirobifluorene moiety. For example, Figure 3 shows the correlation between the imine protons of both diastereomers and their carbon nucleus in complex **Rh10**. In addition, the C=O stretching band disappears at the IR spectra, and a new absorption band at around 1600 cm^−1^ is observed corresponding to the C=N stretching mode. 

## 3. Experimental Section

### 3.1. Materials and Methods

All experiments were carried out under an atmosphere of argon by Schlenk techniques. Solvents were dried by the usual procedures [28] and, prior to use, distilled under argon. All reagents were obtained from commercial sources. The starting material [MCp*(*µ*-Cl)Cl]_2_ (M = Ir, Rh) was prepared as described in the literature [29]. Unless stated, NMR spectra were recorded in CD_2_Cl_2_ or CDCl_3_ at room temperature on a Bruker ARX–400 instrument with resonating frequencies of 400 MHz (^1^H) and 100 MHz (^13^C{^1^H}) using the solvent as the internal lock. ^1^H and ^13^C{^1^H} signals are referred to as internal TMS; downfield shifts (expressed in ppm) are considered positive. ^1^H and ^13^C{^1^H} NMR (or JMOD, J-modulated spin echo experiment) signal assignments were confirmed by {^1^H, ^1^H} COSY, {^1^H, ^1^H} NOESY, {^1^H,^13^C} HSQC, {^1^H,^13^C} HMBC, and/or DEPT experiments. Coupling constants are given in hertz. Mass spectra are referred to the most abundant isotopes and they were acquired using an Apex–Qe or a SolariX XR spectrometer by a high or low-resolution electrospray technique. IR spectra were measured on a Jasco FT/IR-6100 instrument. All the 1D NMR spectroscopy experiments and a comparison of some IR spectra can be found at the Supplementary Materials. 

### 3.2. Synthesis of the Organic Compounds

#### 3.2.1. Synthesis of 2-(9,9′-spirobi[fluoren]-2-yl)-4,4,5,5-tetramethyl-3,2-dioxa-1-boronate (**1**)

To a solution of 2-bromo-9,9′-spirobifluorene (1.0 g, 2.54 mmol) in air-free THF (26 mL), KOAc (746 mg, 7.61 mmol), bis(pinacolato)diborane (965 mg, 3.80 mmol) and PdCl_2_(1,1’-bis(diphenylphosphino)ferrocene)·CH_2_Cl_2_ (204 mg, 0.25 mmol) were added and it was heated at 363 K for 24 h. After that, the reaction mixture was cooled down to room temperature, the solution filtered through SiO_2_ and the product extracted with AcOEt. The crude product was purified through a chromatographic column (SiO_2_, hexane/AcOEt 85/15 to 70/30), affording **1** as a pale yellow solid (1.07 g, 2.41 mmol, 95%). This synthesis is based on the one found in the literature [30].


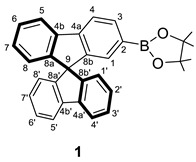


**^1^H NMR** (400 MHz, CD_2_Cl_2_) *δ* 7.91 (dt, *J* = 7.8, 0.9 Hz, 1H, H4 or H5), 7.90 (dt, *J* = 7.7, 1.0 Hz, 2H, H4′ + H5′), 7.89 (dd, *J* = 7.6, 0.7 Hz, 1H, H5 o H4), 7.80 (dd, *J* = 7.6,1.1 Hz, 1H, H3), 7.40 (td, *J* = 7.5, 1.1 Hz, 2H, H3′ + H6′), 7.39 (td, *J* = 7.5, 1.1 Hz, 1H, H6), 7.14 (td, *J* = 7.5, 1.1 Hz, 1H, H7), 7.13 (td, *J* = 7.5, 1.1 Hz, 2H, H2′ + H7′), 7.03 (t, *J* = 0.8 Hz, 1H, H1), 6.69 (dt, *J* = 7.6, 1.0 Hz, 1H, H8), 6.67(dt, *J* = 7.6, 0.9 Hz, 2H, H1′ + H8′), 1.23 (s, 12H, (OC*H*_3_)_4_) ppm.

**^13^C{^1^H} NMR** (101 MHz, CD_2_Cl_2_) *δ* 149.9 (C8a or C8b), 149.2 (C8a’ + C8b’), 148.6 (C8a or C8b), 145.3 (C4a), 142.5 (C4a’ + C4b’), 142.1 (C4b), 135.0 (C3), 130.2 (C1), 129.0 (C6), 128.4 (C7 + C6′ + C7′ + C2′ + C3′), 124.4 (C8), 124.4 (C1′ + C8′), 121.2 (C4 or C5), 120.8 (C5′ + C4′), 120.1 (C5 or C4), 84.3 (Cquat, Bpin), 66.5 (C9), 25.2 (Bpin) ppm. C2 was not observed due to the boron atom effects.

**ESI HRMS**: *m*/*z* calculated for C_31_H_28_BO_2_ [M + H]^+^ 443.2166, found 443.2182.

#### 3.2.2. Synthesis of 2-(9,9′-spirobi[fluoren]-2-yl)pyridine (**2**)

To a solution of **1** (1.43 g, 3.23 mmol) in toluene (35 mL), the following reactants were added in order: Pd(PPh_3_)_4_ (368.6 mg, 0.32 mmol), K_2_CO_3(aq)_ (20 mL, 1 M in water), and 2-bromopyridine (0.46 mL, 4.85 mmol). If nondegassed solvents were used, the reaction mixture was purged with N_2_ for 30 min. Then, the reaction was heated at 363 K for 24 h, and the formation of two yellow phases was observed. After cooling down the reaction mixture, the aqueous phase was extracted three times with CH_2_Cl_2_ and the organic phases were combined and washed twice with H_2_O and once with saturated NaCl. The resultant solution was dried with Na_2_SO_4,_ and the volatiles were removed under vacuum. The crude product was purified through a chromatographic column (SiO_2_, hexane/AcOEt 95/5 to 85/15), affording **2** as a pale yellow solid (1.08 g, 2.74 mmol, 85%). This synthesis is based on the one found in the literature using the SBF-B(OH)_2_ derivative as a starting compound [31].


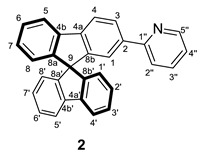


**^1^H NMR** (400 MHz, CD_2_Cl_2_) *δ* 8.52 (dt, *J* = 4.9, 1.3 Hz, 1H, H5′’), 8.06 (dd, *J* = 8.1, 1.6 Hz, H, H3), 7.99 (d, *J* = 8.0 Hz, 1H, H4), 7.9–7.8 (m, 3H, H5 + H4′ + H5′), 7.7–7.6 (m, 2H, H2′’ + H3′’), 7.5–7.4 (m, 4H, H1 + H6 + H3′ + H6’), 7.2–7.1 (m, 4H, H7 + H2′ + H7′ + H4′’), 6.76 (dd, *J* = 7.6, 1.0 Hz, 2H, H1′ + H8′), 6.73 (dd, *J* = 7.6, 1.0 Hz, 1H, H8) ppm.

**^13^C{^1^H} NMR** (101 MHz, CD_2_Cl_2_) *δ* 157.2 (C1′’), 150.0 (C5′’), 149.9 (3C, C8b + C8a’ + C8b’), 149.3 (C8a), 143.4 (C4a), 143.0 (2C, C4a’ + C4b’), 142.4 (C4b), 139.92 (C2), 137.0 (C3′’), 128.5 (C7), 128.4 (5C, C6 + C2′ + C3′ + C6′ + C7′), 127.1 (C3), 124.5 (C1′ + C8′), 122.8 (C1), 122.6 (C4′’), 121.1 (C2′’), 121.0 (C4), 120.9 (3C, C4′ + C5′ + C2′’), 120.7 (C5), 66.6 (C9) ppm. 

**ESI HRMS**: *m*/*z* calculated for C_30_H_20_N [M + H]^+^ 394.1577, found 394.1590.

#### 3.2.3. Synthesis of (±) 2′-(pyridin-2-yl)-9,9′-spirobifluorene-2-carbaldehyde (**3**) 

A solution of **2** (200 mg, 0.51 mmol) in CH_2_Cl_2_ (10 mL) was stirred at 273 K for 5 min. Then, TiCl_4_ (289 mg, 1.53 mmol) was added dropwise and stirred for 30 min at 273 K, leading to a purple solution. After that, CH_3_OCHCl_2_ (176 mg, 1.53 mmol) was added, and the solution was warmed to 298 K overnight. After the reaction time, water was added at 273 K, the product was extracted three times with CH_2_Cl_2,_ and the organic phases were combined and washed twice with H_2_O and once with saturated NaCl. The resultant solution was dried with Na_2_SO_4,_ and the volatiles were removed under vacuum. The crude product was purified through a chromatographic column (SiO_2_, hexane/AcOEt 80/20 to 70/30), leading to a pale yellow solid after removing the volatiles (160 mg, 0.38 mmol, 74%).


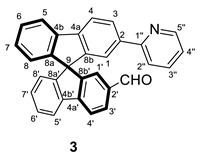


**^1^H NMR** (400 MHz, CD_2_Cl_2_) *δ* 9.84 (s, 1H, C*H*O), 8.58 (dt, *J* = 4.9, 1.0, 0.6 Hz, 1H, H5′’), 8.10 (dd, *J* = 8.0, 1.6 Hz, 1H, H3′), 8.02 (apparent t, *J* = 8.5 Hz, 2H, H5 + H5′), 8.0–7.9 (m, 3H, H3 + H4 + H4′), 7.64 (t, *J* = 7.7 Hz, 1H, H3′’), 7.59 (dd, *J* = 8.0, 1.2 Hz, 1H, H2′’), 7.5–7.4 (m, 3H, H6 + H6′ + H1′), 7.32 (s, 1H, H1), 7.23 (td, *J* = 7.5, 1.1 Hz, 1H, H7), 7.2–7.1 (m, *J* = 6.9 Hz, 2H, H4′’ + H7′), 6.86 (d, *J* = 7.7 Hz, 1H, H8′), 6.73 (d, *J* = 7.6 Hz, 1H, H8) ppm.

**^13^C{^1^H} NMR** (101 MHz, CD_2_Cl_2_) *δ* 191.8 (*C*HO), 157.1 (C1′’), 150.1 (C8b), 149.7 (C8b’), 149.6 (C8a), 148.5 (C5′’), 148.2 (C8a’), 148.2 (C2′), 143.0 (C4a), 141.4 (C4b’), 140.4 (C4b), 139.4 (C4a’), 136.8 (C3′’), 136.2 (C2), 130.5 (C3), 129.7 (C7), 128.4 (C6 + C6′), 128.3 (C7′), 127.4 (C3′), 125.6 (C1′), 124.6 (C8), 124.0 (C8′), 122.6 (C1), 122.1 (C4′’), 121.4 (C2′’), 120.8, 120.7, 120.65 and 120.60 (C5 + C5′ + C4 + C4′), 66.0 (C9) ppm.

**ESI HRMS**: *m*/*z* calculated for C_31_H_20_NO [M + H]^+^ 422.1527, found 422.1539.

**IR**: *ν* 3048 (w, C-H aldehyde), 1686 (s, C=O) cm^−1^.

#### 3.2.4. Synthesis of (±) 7-(4,4,5,5-tetramethyl-3,2-dioxa-1-boronate)-9,9′-spirobifluorene-2,2′-dicarbaldehyde (**4**)

A solution of 2-bromo-9,9′-spirobifluorene (300 mg, 0.75 mmol) in CH_2_Cl_2_ (3 mL) was stirred at 273 K for 5 min. Then, TiCl_4_ (0.33 mL, 3 mmol) was added dropwise and stirred for 30 min at 273 K, leading to a purple solution. After that, CH_3_OCHCl_2_ (0.27 mL, 3 mmol) was added, and the solution was warmed to 298 K overnight. After the reaction time, water was added at 273 K, the product was extracted three times with CH_2_Cl_2,_ and the organic phases were combined and washed twice with H_2_O and once with saturated NaCl. The resultant solution was dried with Na_2_SO_4,_ and the volatiles were removed under vacuum.

The solid obtained in the previous step was dissolved without further purification (0.75 mmol) in air-free THF (7.5 mL) and KOAc (441.6 mg, 4.50 mmol), bis(pinacolato)diborane (286 mg, 1.12 mmol), and PdCl_2_(1,1’-bis(diphenylphosphino)ferrocene)·CH_2_Cl_2_ (61 mg, 0.075 mmol) were added. The reaction mixture was heated at 363 K for 24 h. After that, the reaction mixture was cooled down to room temperature, the solution filtered through SiO_2,_ and the product extracted with AcOEt. The crude product was purified through a chromatographic column (SiO_2_, hexane/AcOEt 85/15 to 70/30), affording **4** as a pale yellow solid (160 mg, 0.32 mmol, 43%).


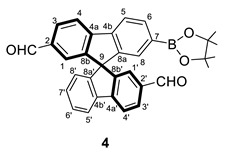


**^1^H NMR** (400 MHz, CDCl_3_) *δ* 9.82 and 9.81 (both s, 1H, C*H*O), 8.04 (dbr, *J* = 8.0 Hz, 1H, H4 or H4′), 8.02 (dbr, *J* = 8.6 Hz, 1H, H4 or H4′), 7.98–7.94 (m, *J* = 7.7, 1.0 Hz, 3H, H5 + H5′ + H6), 7.92 (dd, *J* = 7.9, 1.5 Hz, 1H, H3 or H3′), 7.91 (dd, *J* = 7.9, 1.5 Hz, 1H, H3 or H3′), 7.43 (td, *J* = 7.6, 0.9 Hz, 1H, H6′), 7.24–7.20 (m, 3H, H1 + H1′ + H8) 7.19 (td, *J* = 7.5, 1.1 Hz, 1H, H7′), 6.75 (dt, *J* = 7.6, 0.7 Hz, 1H, H8′), 1.26 (s, 12H, (OC*H*_3_)_4_) ppm.

**^13^C{^1^H} NMR** (101 MHz, CDCl_3_) *δ* 191.7 and 191.6 (CHO), 149.5 (C8a or C8a’), 148.9 (C8a’or C8a), 148.6 (C8b or C8b’), 148.2 (C8b or C8b’), 148.0 (C4a or C4a’), 147.6 (C4a or C4a’), 143.2 (C4b’), 140.5 (C4b), 136.5 (C2a or C2a’), 136.2 (C2a or C2a’), 135.5 (C6), 131.2 (C3 or C3′), 131.1 (C3 or C3′), 130.4 (C8), 129.8 (C7′), 128.6 (C6′), 124.8 (C1 or C1′), 124.7 (C1 or C1′), 124.4 (C8′), 121.6 (C5 or C5′), 121.1 (C5 or C5′), 120.8 (C4 or C4′), 120.7 (C4 or C4′), 84.1 (Cquat, Bpin), 65.6 (C9), 24.9 (Bpin) ppm. C7 was not observed due to the boron atom effects.

#### 3.2.5. Synthesis of (±) 7′-(pyridin-2-yl)-9,9′-spirobifluorene-2,2′-dicarbaldehyde (**5**)

To a solution of **4** (160 mg, 0.32 mmol) in toluene (3 mL), the following reactants were added in order: 2-bromopyridine (0.045 mL, 0.48 mmol), Pd(PPh_3_)_4_ (37 mg, 0.032 mmol), and K_2_CO_3(aq)_ (1.6 mL, 1 M in water). If nondegassed solvents were used, the reaction mixture was purged with N_2_ for 30 min. Then, the reaction was heated at 353 K for 48 h, and the formation of two yellow phases was observed. After cooling down the reaction mixture, the aqueous phase was extracted three times with CH_2_Cl_2_ and the organic phases were combined and washed twice with H_2_O and once with saturated NaCl. The resultant solution was dried with Na_2_SO_4,_ and the volatiles were removed under vacuum. The crude product was purified through a chromatographic column (SiO_2_, hexane/AcOEt 75/25), affording **5** as a pale yellow solid (105 mg, 0.23 mmol, 73%).


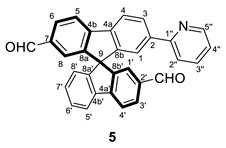


**^1^H NMR** (400 MHz, CDCl_3_) *δ* 9.85 and 9.82 (both s, 1H, C*H*O), 8.57 (dm, *J* = 4.7 Hz, H5′’), 8.11 (dd, *J* = 8.2, 1.4 Hz, H6), 8.10–8.04 (m, 2H, H5 + H4 or H4′), 8.02 (dbr, *J* = 8.0 Hz, 1H, H4 or H4′), 7.98–7.90 (m, *J* = 7.7, 1.0 Hz, 3H, H3 + H3′ + H5′), 7.65 (td, *J* =7.3, 1.6 Hz, 1H, H3′’), 7.59 (dbr, *J* = 7.9 Hz, H2′’), 7.45 (d, *J* = 1.1 Hz, 1H, H8), 7.43 (tbr, *J* = 7.8 Hz, 1H, H6′), 7.30 (d, *J* = 0.8 Hz, 1H, H1 or H1′), 7.25 (d, *J* = 1.1 Hz, 1H, H1 or H1′), 7.20 (tbr, *J* = 7.7 Hz, 1H, H7′), 7.15 (dd, *J* = 7.5, 5.1 Hz, 1H, H4′’), 6.86–6.77 (m, 1H, H8′) ppm.

**^13^C{^1^H} NMR** (101 MHz, CDCl_3_) *δ* 191.6 (both CHO), 156.5 (C1′’), 149.5 (C5′’), 149.6 (C8b or C8b’), 149.4 (C8b’or C8b), 148.7 (C8a or C8a’), 148.5 (C8a or C8a’), 148.1 (C4a or C4a’), 147.4 (C4a or C4a’), 141.2 (C7), 140.8 (C4b’), 140.4 (C4b), 136.3 (C2a or C2a’), 136.2 (C2a or C2a’), 136.9 (C3′’), 131.22 (C3 or C3′), 131.18 (C3 or C3′), 129.8 (C6), 128.6 (C7′), 127.7 (C6′), 124.8 (C1 or C1′), 124.6 (C1 or C1′), 124.3 (C8′), 122.8 (C8), 122.5 (C4′’), 121.8 (C5′), 121.5 (C4 or C4′), 121.01 (C5 or C4 or C5′), 120.97 (C5 or C4 or C4′), 120.8 (C2′’), 65.7 (C9) ppm. 

**ESI HRMS**: *m*/*z* calculated for C_32_H_20_NO_2_ [M + H]^+^ 450.1494, found 450.1478.

**IR**: *ν* 2961 (w, C-H aldehyde), 1689 (s, C=O) cm^−1^.

### 3.3. Synthesis of the Organometallic Complexes

#### 3.3.1. Synthesis of [MCp*Cl(2-py-SBF)] (M = Rh (**Rh6**), Ir (**Ir6**)), and [MCp*(OCOCH_3_)(2-py-SBF)] (M = Rh (**Rh7**), Ir (**Ir7**))

[MCp*Cl(*µ*-Cl)]_2_ (M = Ir, Rh) (96 mg for Ir and 74 mg for Rh, 0.12 mmol) were dissolved in CH_2_Cl_2_ (15 mL), and **2** (100 mg, 0.25 mmol) was added, followed by KOAc (30 mg, 0.30 mmol). After stirring for 24 h at room temperature, the color changed from orange to yellow (for Ir) or from red to orange (for Rh), and the solution was filtrated through Celite^®^. Then, the volatiles were removed by vacuum. Later, pentane (3 × 5 mL) was added. For the rhodium complexes, products were washed with pentane, while for iridium, a partial extraction of them with pentane was achieved. In the latter case, the volatiles of pentane solution were, again, removed by vacuum, leading to a mixture of [IrCp*Cl(2-py-SBF)] (**Ir6**) and [IrCp*(OCOCH_3_)(2-py-SBF)] (**Ir2**) in a 1:3 ratio. Meanwhile, the solid that precipitates with pentane was dried in a vacuum, giving a mixture of [MCp*Cl(2-py-SBF)] (M = Rh (**Rh6**), Ir (**Ir6**)) and [MCp*(OCOCH_3_)(2-py-SBF)] (M = Rh (**Rh7**), Ir (**Ir7**)) in a 1:1.5 ratio for both Ir and Rh complexes. Crystals of **Ir6**, **Ir7,** and **Rh6** were obtained from the slow evaporation of a CH_2_Cl_2_:hexane mixture. Isolated mass of **Ir6** + **Ir7**: 168 mg. Isolated mass of **Rh6** + **Rh7**: 149 mg.

**ESI HRMS**: *m*/*z* calculated for C_40_H_33_NIr [M-Cl or OAc]^+^ 720.2242, found 720.2218.


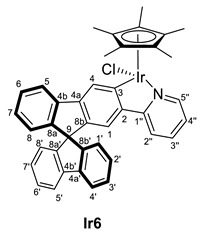


**^1^H NMR (400 MHz, CD_2_Cl_2_)***δ* 8.64 (dt, *J* = 5.7, 1.3 Hz, 1H, H5′’), 8.34 (d, *J* = 0.6 Hz, 1H, H4), 8.01 (dt, *J* = 7.6, 1.0 Hz, 1H, H5), 7.905 (dt, *J* = 7.6, 1.0 Hz, 1H, H5′), 7.897 (dt, *J* = 7.7, 0.9 Hz, 1H, H4′), 7.53–7.46 (m, 2H, H2′’ + H3′’), 7.43–7.36 (m, 3H, H6 + H6′ + H3′), 7.16–7.09 (m, 3H, H7 + H2′ + H7′), 7.05 (d, *J* = 0.6 Hz, 1H, H1), 7.03 (ddd, *J* = 6.3, 5.8, 2.5 Hz, 1H, H4′’), 6.80 (dt, *J* = 7.6, 0.9 Hz, 1H, H8′), 6.79 (dt, *J* = 7.6, 0.9 Hz, 1H, H1′), 6.68 (dt, *J* = 7.6, 0.9 Hz, 1H, H8), 1.74 (s, 15H, C_5_(CH_3_)_5_) ppm.

**^13^C{^1^H} NMR (101 MHz, CD_2_Cl_2_)***δ* 167.0 (C1′’), 164.1 (C3), 152.0 (C5′’), 150.5 and 150.2 (C8a’ + C8b’), 149.9 (C8a), 145.0 (C8b), 144.7 (C2), 143.0 (C4a), 142.5 and 142.4 (C4a’ + C4b’), 142.2 (C4b), 137.4 (C3′’), 128.6, 128.3, 128.2 and 128.1 (C2′ + C7′ + C3′ + C6′ + C6 + C7), 127.6 (C4), 124.8 and 124.3 (C8′ + C1′) and 124.2 (C8), 122.7 (C4′’), 121.1 (C5), 120.7 and 120.5 (C4′ + C5′), 119.5 (C1), 119.4 (C2′’), 89.3 (*C*_5_(CH_3_)_5_), 66.1 (C9), 9.3 (C_5_(*C*H_3_)_5_). 


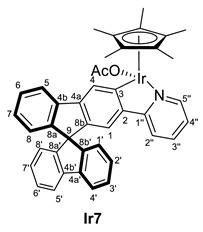


**^1^H NMR (400 MHz, CD_2_Cl_2_)***δ* 9.07 (d, *J* = 5.3 Hz, 1H; H5′’), 8.54 (s, 1H, H4), 8.03 (d, *J* = 8.0 Hz, 1H, H5), 7.90 (d, *J* = 7.9 Hz, 2H, H4′ + H5′), 7.52–7.34 (m, 5H, H2′’ + H3′’+ H6′ + H3′ + H6), 7.19–7.08 (m, 3H, H7 + H7′ + H2′), 7.01 (s, 1H, H1), 7.07–6.96 (m, 1H, H4′’), 6.78 (d, *J* = 7.6 Hz, 2H, H8′ + H1′), 6.68 (d, *J* = 7.3 Hz, 1H, H8), 1.71 (s, 15H, C_5_(CH_3_)_5_), 1.66 (s, 3H OCOCH_3_) ppm.

**^13^C{^1^H} NMR (101 MHz, CD_2_Cl_2_)***δ* 177.2 (O*C*OCH_3_), 166.8 (C1′’), 164.4 (C3), 153.37 (C5′’), 149.8 and 149.4 (C8a’ + C8b’), 149.3 (C8a), 145.3 (C2), 143.7 (C8b), 142.7 (C4b), 141.9 and 141.8 (C4a’ + C4b’), 141.6 (C4a), 136.9 (C3′’), 127.9, 127.7 and 127.6 (C6 + C7 + C6′ + C7′ + C2′ + C3′), 127.0 (C4), 124.0 and 123.7 (C8′ + C1′), 123.6 (C8), 121.1 (C4′’), 120.0 (C4′ + C5′), 118.8 (C1), 118.2 (C2′’), 87.2 (*C*_5_(CH_3_)_5_), 65.6 (C9), 23.1 (OCO*C*H_3_), 8.8 (C_5_(*C*H_3_)_5_) ppm. 


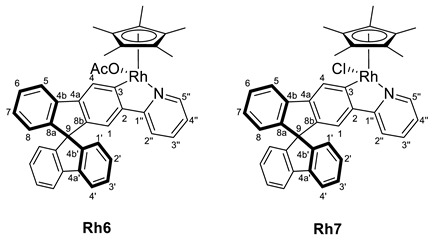


**^1^H NMR (400 MHz, CD_2_Cl_2_)***δ* 8.73 (d br, *J* = 5.6 Hz, 2H, H5′’), 8.43–8.36 (m, 2H, H4), 8.09 (d, *J* = 7.6 Hz, 2H, H5), 8.04–7.90 (m, 4H, H4′), 7.66–7.57 (m, 2H, H3′’), 7.55–7.38 (m, 8H, H2′’ + H3′ + H6), 7.30–7.10 (m, 8H, H7+ H4′’ + H2′), 7.05 (s, 2H, H1), 6.92–6.82 (m, 4H, H1′), 6.75–6.67 (m, 2H, H8), 2.00 (s, 3H OCOCH_3_), 1.72 (s br, 30H, C_5_(CH_3_)_5_) ppm.

**^13^C{^1^H} NMR (101 MHz, CD_2_Cl_2_)** Complex **Rh6:** *δ* 179.0 (d, ^1^*J*_CRh_ = 32 Hz, C3), 164.6 (C1′’), 151.4 (C5′’), 150.3–149.0 (C8a + C8b + C4b’), 143.7 (C4a), 143.3 (d, ^2^*J*_CRh_ = 3 Hz, C2), 141.9–141.7 (C4b + C4a’), 136.9 (C3′’), 128.5–127.5 (C6 + C7 + C2′ + C3′ + C4), 124.3 (C1′), 123.8 (C8), 121.9 (C4′’), 120.5 (C5), 120.0 (C4′), 119.1 (C2′’), 118.5 (C1), 96.1 (d, ^1^*J*_CRh_ = 4 Hz, *C*_5_(CH_3_)_5_), 65.63 (C9), 9.0 (C_5_(*C*H_3_)_5_) ppm. Complex **Rh7:** *δ* 178.5 (br, O*C*OCH_3_), 179.4 (d, ^1^*J*_CRh_ = 33 Hz, C3), 164.7 (C1′’), 151.4 (C5′’), 150.3–149.0 (C8a + C8b + C4b’), 143.5 (C4a), 143.3 (d, ^2^*J*_CRh_ = 3 Hz, C2), 141.9–141.7 (C4b + C4a’), 137.0 (C3′’), 128.5–127.5 (C6 + C7 + C2′ + C3′ + C4), 124.3 (C1′), 123.7 (C8), 122.0 (C4′’), 120.7 (C5), 120.1 (C4′), 119.1 (C2′’), 118.5 (C1), 96.2 (d, ^1^*J*_CRh_ = 4 Hz, *C*_5_(CH_3_)_5_), 65.6 (C9), 22.0 (OCO*C*H_3_), 9.0 (C_5_(*C*H_3_)_5_) ppm. 

#### 3.3.2. Synthesis of (*R,M*)/(*R,P*)-[MCp*Cl(2‘-CHO-2-py-SBF)] (M = Rh (**Rh8**), Ir (**Ir8**)) 

[MCp*Cl(*µ*-Cl)]_2_ (M = Ir, Rh) (96 mg for Ir and 74 mg for Rh, 0.12 mmol) were dissolved in CH_2_Cl_2_ (10 mL), and **3** (100 mg, 0.24 mmol) was added, followed by KOAc (30 mg, 0.30 mmol). After stirring for 24 h at room temperature, the color changed from orange to brownish (for Ir) or from red to orange (for Rh), and the solution was filtrated through a SiO_2_ pad using CH_2_Cl_2_/MeOH 95/5 as eluent. Then, the volatiles were removed by vacuum, giving the complex (*R,M*)/(*R,P*)-[MCp*Cl(2‘-CHO-2-py-SBF)] (M = Rh (**Rh8**), Ir (**Ir8**)) as a mixture of diastereoisomers A and B in a 60:40 (for **Rh8**) and 63:37 ratio (for **Ir8**), respectively. Unfortunately, A and B cannot be assigned to a specific diastereoisomer (*R,M*)* or (*R,P*)*. Yield for **Rh8**: 150 mg (0.11 mmol, 90%). Yield for **Ir8**: 178 mg (0.11 mmol, 95%).


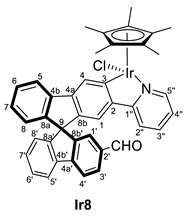


**^1^H NMR (400 MHz, CDCl_3_)** *δ* 9.88 (s, 1H, CHO isomer A), 9.75 (s, 1H, isomer B), 8.68 (d pseudo t, *J* = 5.7, 1.1 Hz, 2H, H5′’AB), 8.38 (s, 1H, H4B), 8.37 (s, 1H, H4A), 8.03–7.90 (m, 8H, H5AB + H5′AB + H4′AB + H3′AB), 7.49–7.45 (m, 4H, H2′’AB + H3′’AB), 7.44- 7.38 (m, 4H, H6AB + H6′AB), 7.43 (s, partially overlapped, 1H, H1′A), 7.25 (d, *J* = 1.1 Hz, 1H, H1′B), 7.23 (td, *J* = 7.6, 1.1 Hz, 1H, H7′B), 7.19 (td, *J* = 7.5, 1.1 Hz, 1H, H7′A), 7.12 (td, *J* = 7.1, 1.1 Hz, 1H, H7B), 7.11 (td, *J* = 7.5, 1.1 Hz, 1H, H7A), 7.03 (s, 1H, H1B), 7.01 (s, 1H, H1A), 7.00–6.96 (m, 2H, H4′’AB), 6.93 (dt, *J* = 7.6, 0.9 Hz, 1H, H8′B), 6.81 (dt, *J* = 7.7, 0.9 Hz, 1H, H8′A), 6.71 (d, *J* = 7.8 Hz, 1H, H8B), 6.69 (d, *J* = 7.8 Hz, 1H, H8A), 1.78 (s, 15H, C_5_(CH_3_)_5_ isomer A), 1.77 (s, 15H, C_5_(CH_3_)_5_ isomer B) ppm. 

**^13^C{^1^H} NMR (101 MHz, CDCl_3_)** *δ* 192.2 (CHO isomer A), 191.9 (CHO isomer B), 166.8 (C3A), 166.7 (C3B), 163.9 (C1′’B), 163.8 (C1′’A), 151.4 (C5′’AB), 151.1 (C8a’B), 151.0 (C8a’A), 150.8 (C8b’A), 150.3 (C8b’B), 148.7 (C4a’AB), 148.3 (C8aA), 147.5 (C8aB), 144.54 (C4aA), 144.47 (C4aB), 144.4 (C8bB), 144.3 (C8bA), 142.3 (C2AB), 141.3 (C4bAB), 140.5 (C4b’B), 139.9 (C4b’A), 136.9 (C3′’AB), 136.2 (C2′B), 136.0 (C2′A), 130.9 (C3′A), 129.9 (C7′A), 129.3 (C7′B), 128.2 (C3′B), 128.03 + 128.00 + 127.99 + 127.95 (C6AB + C6′AB + C7AB), 127.9 (C1′B), 127.2 (C4AB), 125.2 (C8′A), 125.1 (C1′A), 124.6 (C8′B), 123.9 (C8B), 123.8 (C8A), 122.2 (C4′’B), 122.1 (C4′’A), 121.2 + 120.7 + 120.4 (C4′B + C5B + C5′B), 121.0 + 120.8 + 120.3 (C4′A + C5A + C5′A), 119.4 (C1A), 119.3 (C1B), 119.11 (C2′’B), 119.07 (C2′’A), 88.84 (*C*_5_(CH_3_)_5_, isomer A), 88.76 (*C*_5_(CH_3_)_5_, isomer B), 65.5 (C9AB), 9.2 (C_5_(*C*H_3_)_5_, isomer A), 9.1 (C_5_(*C*H_3_)_5_, isomer B).

**ESI HRMS**: *m*/*z* calculated for C_41_H_33_ONIr [M-Cl]^+^ 748.2191, found 748.2174.

**IR**: *ν* 1738 (s, C=O) cm^−1^.


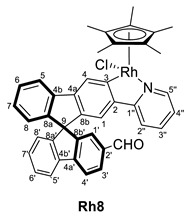


**^1^H NMR (400 MHz, CD_2_Cl_2_)** *δ* 9.86 (s, 1H, CHO isomer A), 9.77 (s, 1H, isomer B), 8.70 (d, *J* = 5.6, 2H, H5′’AB), 8.370 (s, 1H, H4B), 8.369 (s, 1H, H4A), 8.12–7.91 (m, 6H, H5 + H5′ + H3′AB), 7.64–7.52 (m, 2H, H3′’), 7.50–7.40 (m, 8H, H6 + H6′ + H4′ + H2′’AB), 7.35 (s, 1H, H1′A), 7.30 (s, 1H, H1′B), 7.27–7.19 (m, 2H, H7′AB), 7.19–7.06 (m, 4H, H4′’ + H7AB), 6.98 (s, 2H, H1AB), 6.89–6.80 (m, 2H, H8′AB), 6.74–6.65 (m, 2H, H8AB), 1.70 (s, 15H, C_5_(CH_3_)_5_ isomer A), 1.69 (s, 15H, C_5_(CH_3_)_5_ isomer B) ppm. 

**^13^C{^1^H} NMR (101 MHz, CD_2_Cl_2_)** *δ* 192.0 (CHO isomer A), 191.9 (CHO isomer B), 180.0 (d, ^1^*J*_CRh_ = 30 Hz, C3A), 179.9 (d, ^1^*J*_CRh_ = 30 Hz, C3B), 165.0 (d, ^2^*J*_CRh_ = 2 Hz, C1′’A), 163.8 (d, ^2^*J*_CRh_ = 1 Hz, C1′’B), 151.81 (C5′’B), 151.76 (C5′’A), 151.2 (C8a’B), 151.1 (C8a’A), 150.7 (C8b’A), 150.4 (C8b’B), 149.0 (C4a’AB), 148.0 (C8aB), 148.5 (C8aA), 144.0 (C8bAB), 143.8 (C4aB), 142.6 (d, ^2^*J*_CRh_ = 3 Hz, C2AB), 142.5 (C4bAB), 140.4 (C4b’B), 136.9 (C3′’AB), 136.7 (C2′B), 136.5 (C2′A), 131.1 (C3′AB), 130.1 (C7′B), 129.6 (C7′A), 128.9–128.2 (C6 + C6′ + C7 + C4′AB), 127.1 (C1′B), 128.8 (d, ^3^*J*_CRh_ = 2 Hz, C4A), 128.7 (d, ^3^*J*_CRh_ = 2 Hz, C4B), 124.9 (C1′A), 124.5 (C8′AB), 124.0 (C8AB), 122.3 (C4′’AB), 121.7 -120.7 (C5 + C5′AB), 119.4 (C2′’AB), 118.8 (C1AB), 96.62 (d, ^1^*J*_CRh_ = 4 Hz, *C*_5_(CH_3_)_5_, isomer B), 96.56 (d, ^1^*J*_CRh_ = 4 Hz, *C*_5_(CH_3_)_5_, isomer A), 65.9 (C9B), 65.8 (C9A), 9.45 (C_5_(*C*H_3_)_5_, isomer A), 9.41 (C_5_(*C*H_3_)_5_, isomer B).

**ESI HRMS**: *m*/*z* calculated for C_41_H_33_ONRh [M-Cl]^+^ 658.16116, found 658.16324.

**IR**: *ν* 1691 (s, C=O) cm^−1^.

#### 3.3.3. Synthesis of (*R,M*)/(*R,P*)-[IrCp*Cl(2‘,7-diCHO-2-py-SBF)] (**Ir9**) 

[IrCp*Cl(*µ*-Cl)]_2_ (88 mg, 0.11 mmol) was dissolved in CH_2_Cl_2_ (10 mL), and **5** (100 mg, 0.22 mmol) was added, followed by KOAc (23 mg, 0.23 mmol). After stirring for 24 h at room temperature, the color changed from orange to brownish, and the solution was filtrated through a SiO_2_ pad using CH_2_Cl_2_/MeOH 95/5 as an eluent. Then, the volatiles were removed by vacuum, giving the complex (*R,M*)/(*R,P*)-[IrCp*Cl(2‘,7-diCHO-2-py-SBF)] (**Ir9**) as a mixture of diastereoisomers A and B in a 58:42 ratio, respectively. Unfortunately, they could not be assigned to the specific isomer (*R,M*)* or (*R,P*)*. Yield 166 mg (0.20 mmol, 93%).


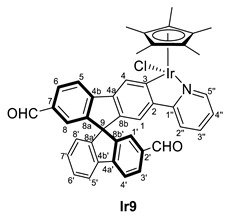


**^1^H NMR (400 MHz, CDCl_3_)** *δ* 9.87 and 9.82 (both s, 1H, CHO isomer A), 9.83 and 9.73 (both s, 1H, isomer B), 8.68–8.64 (m, 2H, H5′’AB), 8.43 (s, 1H, H4B), 8.42 (s, 1H, H4A), 8.43 (d, *J* = 7.8 Hz, 1H, H4′B or H5B), 8.09 and 8.08 (both d, *J* = 7.9 Hz, 1H, H4′A and H5A), 8.00–7.87 (m, 7H, H6AB + H5′AB + H3′AB+ H4′B or H5B), 7.51–7.47 (m, 5H, H2′’AB +H3′’AB + H1′B), 7.44 (td, *J* = 7.6, 1.0 Hz, 1H, H6′B), 7.41 (td, *J* = 7.6, 1.0 Hz, 1H, H6′A), 7.37 (d, *J* = 1.1H Hz, 1H, H1′A), 7.22 (td, *J* = 7.7, 1.2 Hz, 1H, H7′B), 7.20 (d, *J* = 1.0 Hz, 1H, H8A), 7.19 (d, *J* = 1.4 Hz, 1H, H8B), 7.17 (td, *J* = 7.6, 1.0 Hz, 1H, H7′A), 7.05 (s, 1H, H1B), 7.04 (s, 1H, H1A), 7.03 (d, *J* = 2.7 Hz, 1H, H4′’B), 7.01 (d, *J* = 2.7 Hz, 1H, H4′’A), 6.87 (dt, *J* = 7.6, 0.8 Hz, 1H, H8′B), 6.76 (dt, *J* = 7.6, 0.7 Hz, 1H, H8′A), 1.76 (s, 15H, C_5_(CH_3_)_5_ isomer A), 1.75 (s, 15H, C_5_(CH_3_)_5_ isomer B) ppm. 

**^13^C{^1^H} NMR (101 MHz, CD_2_Cl_2_)** *δ* 192.0–191.8 (CHO both isomers AB), 166.4 and 166.3 (C3AB), 164.5 and 164.4 (C1′’AB), 151.2 (C5′’AB), 150.4–146.4 (several resonances, C8a’AB + C8b’AB + C4a’AB + C8aAB + C4aAB), 143.1–140.6 (several resonances, C8bAB + C2AB + C4bAB + C4b’AB), 137.4 (C3′’AB), 136.5 (C2′AB), 131.6–119.7 (CH signals of SBF moiety), 123.3 (C4′’AB), 119.4 (C2′’AB), 89.3 (*C*_5_(CH_3_)_5_, isomers AB), 65.6 (C9AB), 9.2 (C_5_(*C*H_3_)_5_, isomer A), 9.1 (C_5_(*C*H_3_)_5_, isomer B).

**ESI HRMS**: *m*/*z* calculated for C_42_H_33_O_2_NIr [M-Cl]^+^ 776.2140, found 776.2124.

**IR**: *ν* 1600 and 1690 (m, C=O) cm^−1^.

### 3.4. Reactivity of Complexes **Ir8**, **Rh8** and **Ir9** towards Amines

#### 3.4.1. General Procedure for the Reactivity of (*R,M*)/(*R,P*)-[MCp*Cl(2‘-CHO-2-py-SBF)] (M = Rh (**Rh8**), Ir (**Ir8**)) or (*R,M*)/(*R,P*)-[IrCp*Cl(2‘,7-diCHO-2-py-SBF)] (**Ir9**) with amines

Complex **Rh8, Ir8,** or **Ir9** (0.1 mmol) was dissolved in CHCl_3_ (10 mL) after drying it over activated powder 4Å molecular sieves. Afterwards, an excess of the corresponding amine was added (1.2–4 equiv, 0.12–0.4 mmol), and the solution was stirred overnight. Then, the solution was filtrated through Celite^®^ and volatiles were removed under vacuum. Finally, the brownish solid was dried. In every case, both possible diastereomers are formed.

#### 3.4.2. Selected Characterization Data of Imine Complexes

(*R,M*)/(*R,P*)-[IrCp*Cl(2‘-CH=NPh-2-py-SBF)] (**Ir10**) 


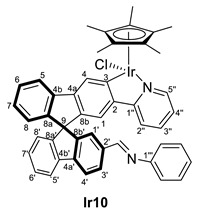


**^1^H NMR (400 MHz, CD_2_Cl_2_)***δ* 8.40 (s, overlapped with other signals, CH=N), 8.29 (s, CH=N) ppm.

**^13^C{^1^H} NMR (101 MHz, CD_2_Cl_2_)** *δ* 164.4 and 164.2 (C3); 160.5 and 160.2 (CH=N); 152.4 (C1′’’); 136.7 (C2′) ppm.

**ESI HRMS**: *m*/*z* calculated for C_47_H_39_N_2_IrCl [M + H]^+^ 858.23472, found 858.23420; calculated for C_47_H_39_N_2_Ir [M + H-Cl]^+^ 825.27189, found 825.28281.

**IR**: *ν* 1601 (m, C=N) cm^−1^.

(*R,M*)/(*R,P*)-[IrCp*Cl(2‘-CH=N(*o*-NH_2_-C_6_H_4_)-2-py-SBF)] (**Ir11**) 


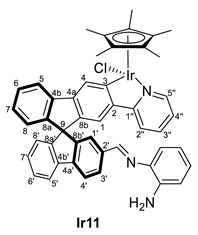


**^1^H NMR (400 MHz, CD_2_Cl_2_)***δ* 8.41 (s, mayor isomer, CH=N), 8.37 (s, minor isomer, CH=N) ppm.

**^13^C{^1^H} NMR (101 MHz, CD_2_Cl_2_)** *δ* 164.1 (br, observed by HMBC NMR experiment, C3); 157.8 (CH=N); 137.2 (C2′) ppm.

**ESI HRMS**: *m/z* calculated for C_47_H_37_N_3_Ir [M-Cl-2H]^+^ 836.25932, found 836.26229.

**IR**: *ν* 1601 (m, C=N) cm^−1^.

(*R,M*)/(*R,P*)-[IrCp*Cl(2‘,7-di(CH=NPh)-2-py-SBF)] (**Ir13**) 


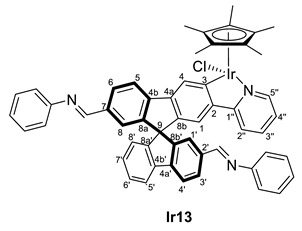


**^1^H NMR (400 MHz, CD_2_Cl_2_)***δ* 8.37, 8.36 (2H) and 8.31 (s, CH=N) ppm.

**^13^C{^1^H} NMR (101 MHz, CD_2_Cl_2_)** *δ* 164.4 and 164.3 (C3); 160.5, 160.3 (2C) and 160.1 (CH=N); 152.4 (3s, C1′’’); 136.8, 136.5 and 136.4 (C7 + C2′) ppm.

**IR**: *ν* 1600 (m, C=N) cm^−1^.

(*R,M*)/(*R,P*)-[RhCp*Cl(2‘-CH=NPh-2-py-SBF)] (**Rh10**) 

In this case, the reaction mixture was refluxed overnight.


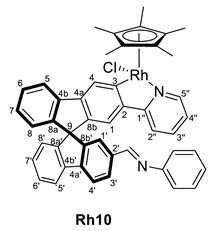


**^1^H NMR (400 MHz, CD_2_Cl_2_)***δ* 8.36 (s, CH=N), 8.28 (s, CH=N) ppm.

**^13^C{^1^H} NMR (101 MHz, CD_2_Cl_2_)** *δ* 179.7 and 179.6 (both d, ^1^*J*_C-Rh_ = 33.2 Hz, C3); 160.5 and 160.2 (CH=N); 152.4 (2s, C1′’’); 136.8 and 136.4 (C2′) ppm.

**IR**: *ν* 1600 (m, C=N) cm^−1^.

(*R,M*)/(*R,P*)-[RhCp*Cl(2‘-CH=N(CH_2_CH_2_NH_2_)-2-py-SBF)] (**Rh12**) 


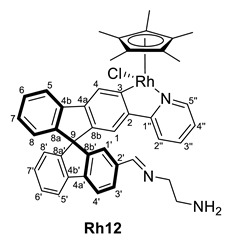


**^1^H NMR (400 MHz, CD_2_Cl_2_)***δ* 8.10 (s, CH=N), 8.03 (s, overlapped with other signals CH=N), 3.70–3-58 (m, 4H, CH_2_) ppm.

**^13^C{^1^H} NMR (101 MHz, CD_2_Cl_2_)** *δ* 179.8 (br, observed by HMBC NMR experiment, C3); 162.1 (CH=N); 136.7 (C2′); 62.2–62.0 (CH_2_ groups) ppm.

**ESI HRMS**: *m*/*z* calculated for C_43_H_38_N_3_Rh [M-HCl]^+^ 699.21206, found 699.19232

**IR**: *ν* 1599 (m, C=N) cm^−1^.

### 3.5. Crystallography

Crystallographic data for complexes **Ir6**, **Ir7,** and **Rh6** were collected on a Bruker D8 Venture Photon 100 CMOS diffractometer at 100 K using Mo-Kα radiation (λ = 0.71073 Å). The frames were integrated with the Bruker SAINT 6.01 [32] software package, and the data were corrected for absorption using the program SADABS-2016/2 [33]. The structures were solved by direct methods using the program SHELXL-2018/3 [34]. All nonhydrogen atoms were refined with anisotropic thermal parameters by full-matrix least-squares calculations on F^2^ using the program SHELXL-2018/3 [35]. Hydrogen atoms were inserted at calculated positions and were constrained with isotropic thermal parameters. For **Ir7**, the high disorder was found in the nonsubstituted SBF branch, which does not allow data publication but supports the structure provided by the rest of the characterization data. The crystal data and structure refinement table can be found at the Supplementary Materials.

## 4. Conclusions

Iridium and rhodium metallacyclic complexes bearing spirobifluorene moiety [MCp*X(2-py-SBF)] (M = Rh, Ir; X = Cl, OAc)(**6**,**7**) have been synthesized and fully characterized. While these complexes cannot react towards amines, the inclusion of one or two aldehyde groups at the spirobifluorene moiety to give complexes **8** and **9** allowed such reactivity. The reaction took place through the condensation reaction of an aldehyde with aromatic or aliphatic amines leading to imine complexes (*R,M*)/(*R,P*)-[MCp*Cl(2‘-CH=NR-2-py-SBF)] (M = Rh, Ir)(**10**–**12**) and (*R,M*)/(*R,P*)-[IrCp*Cl(2‘, 7-di(CH=NPh)-2-py-SBF)](**13**) under mild conditions. Interestingly, the reactivity of these complexes toward each type of amine depends on the transition metal coordinated. Thus, rhodium complex **Rh8** reacts with aliphatic amines, while both **Ir8** and **Ir9** react with aromatic ones. This selectivity could be the grounds for the synthesis of organometallic complexes based on C–N bidentate spirobifluorene ligands for the detection of aliphatic amines with rhodium and aromatic amines with iridium.

## Figures and Tables

**Table 1:** Selected bond lengths (Å) and angles (°) for complex **6**.

Bond	Ir6	Rh6	Angle	Ir6	Rh6
^a^ M-CT	1.823	1.833	C(22)-M(1)-N(1)	77.96(13)	78.83(9)
C(15)-C(21)	1.446(5)	1.459(3)	C(21)-C(22)-M(1)	115.6(2)	115.32(17)
M(1)-Cl(1)	2.3873(9)	2.3881(7)	C(22)-M(1)-Cl(1)	87.92(9)	89.29(7)
C(21)-C(22)	1.413(5)	1.418(3)	N(1)-M(1)-Cl(1)	88.58(8)	91.46(6)
M(1)-C(22)	2.047(3)	2.027(2)	C(15)-N(1)-M(1)	116.8(2)	116.23(16)
M(1)-N(1)	2.097(3)	2.094(2)	N(1)-C(15)-C(21)	114.1(3)	113.9(2)
N(1)-C(15)	1.361(4)	1.358(3)			

^a^ In M-CT, M refers to the Ir or Rh atom, while CT refers to the centroid of the Cp* ligand.

**Table 2:**
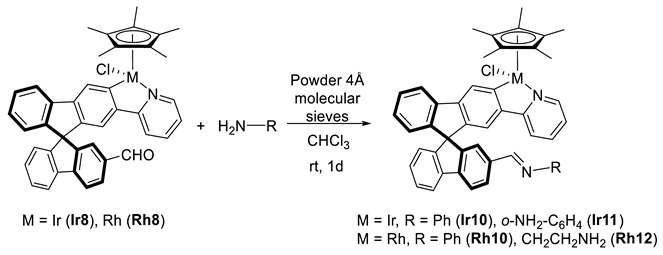
Reactivity of **8** towards amines.

Entry	Metal Complex	R	Complex: Amine Ratio	Temperature (K)	Product	Conversion (%) ^a^
1	**Ir8**	Ph	1:4	298	**Ir10**	99
2	**Ir8**	*o*-NH_2_-C_6_H_4_	1:1.2	298	**Ir11**	98
3	**Ir8**	CH_2_CH_2_NH_2_	1:2	298	**Ir12**	0
4	**Ir8**	CH_2_CH_2_NH_2_	1:2	343	**Ir12**	0
5	**Rh8**	Ph	1:3	298	**Rh10**	0
6	**Rh8**	Ph	1:3	343	**Rh10**	99
7	**Rh8**	CH_2_CH_2_NH_2_	1:2	298	**Rh12**	99

^a^ Conversion of the organometallic complex determined by ^1^H NMR spectroscopy in the presence of excess amine.

**Table 3:** Characteristic NMR and IR data of imine complexes.

Complex	^1^H NMR (ppm) ^a^	^13^C{^1^H} NMR (ppm) ^a^			IR (cm^−1^) ^b^
	***δ* (C*H*=N)**	***δ* (*C*H=N)**	***δ* (*C*-CH=N)**	***δ* (Ir-*C*)**	***ν* (C=N)**
**Ir10**	8.40, 8.29	160.5, 160.2	136.7	164.4, 164.2	1601
**Ir11**	8.41 (major) 8.37 (minor)	157.8	137.2	164.1	1601
**Ir13**	8.37, 8.36 (2H), 8.31	160.5, 160.3 (2C), 160.1	136.8, 136.5, 36.4 (C2′ + C7)	164.4, 164.3	1600
**Rh10**	8.36, 8.28	160.5, 160.2	136.8 and 136.4	179.7, 176.6 (both d, J = 32 Hz)	1600
**Rh12**	8.10, 8.03	162.1	136.7	179.8 (br)	1599

^a^ Measured at 400 MHz in CD_2_Cl_2_, ^b^ In an ATR-diamond IR spectrometer.

## Data Availability

CCDC 2298148-2298149 contain the supplementary crystallographic data for this paper. These data can be obtained free of charge via www.ccdc.cam.ac.uk/data_request/cif (accessed on 30 October 2022), by emailing data_request@ccdc.cam.ac.uk, or by contacting The Cambridge Crystallographic Data Centre, 12 Union Road, Cambridge CB2 1EZ, UK; fax: +44 1223 336033.

## Supplementary Materials

The following supporting information can be downloaded at https://www.mdpi.com/article/10.3390/molecules28207155/s1, Table S1: Crystal data and structure refinement; Figures S1–S30: NMR spectra of synthesized compounds; Figure S31: IR spectra comparison of some Rh complexes.
