# Supplementary material for: Selective Schiff Base Formation of Group 9 Organometallic Complexes with Functionalized Spirobifluorene Ligands
Source: Molecules. 2023 Oct 18;28(20):7155. doi: 10.3390/molecules28207155 (PMC10609629; doi:10.3390/molecules28207155)
Supplement: Supplementary file 1 [file molecules-28-07155-s001.zip › molecules-2667512-supplementary.pdf]

Supporting Information

Selective Schiff base formation of group 9 organometallic complexes with functionalized spirobifluorene ligands

Krystal M. Cid-Seara,[1] Raquel Pereira-Cameselle,[2] Sandra Bolaño[1,*] and Maria Talavera[1,*]

[1] *Universidade de Vigo, Departamento de Química Inorgánica, Campus Universitario, 36310, Vigo, Spain*

[2] *Universidade de Vigo, Departamento de Química Orgánica, Campus Universitario, 36310, Vigo, Spain*

Corresponding authors: bgs@uvigo.gal, matalaveran@uvigo.gal

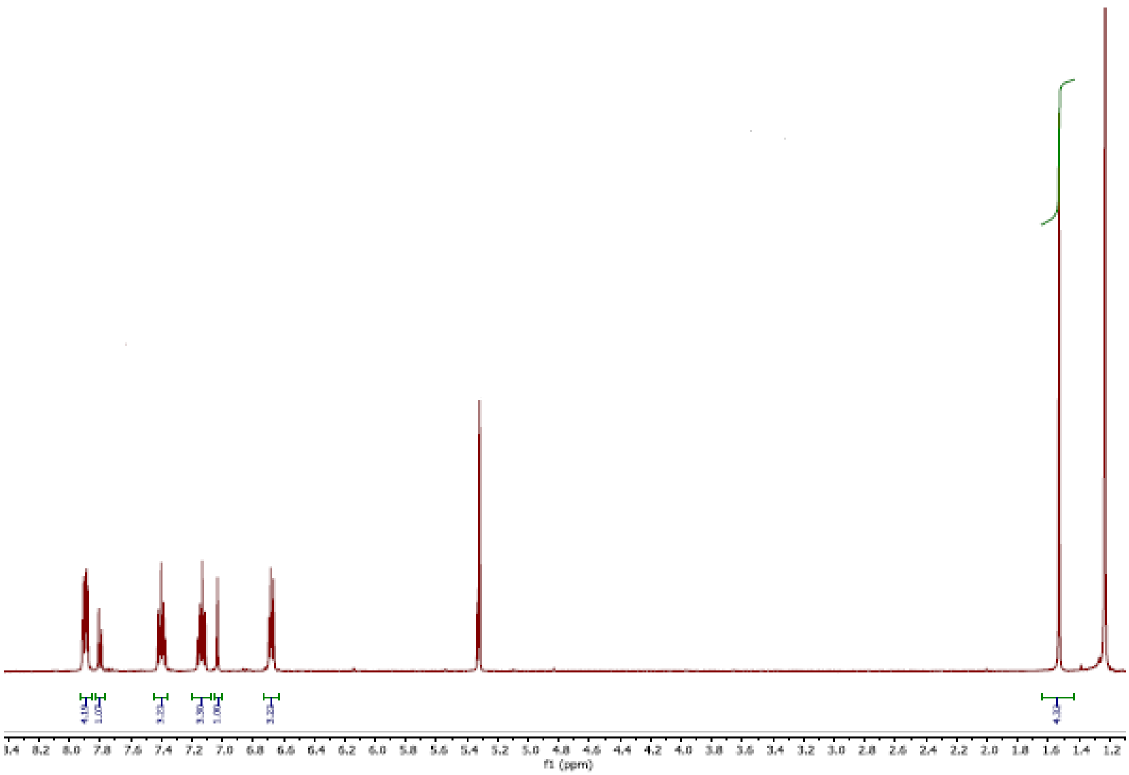

Figure S1. ^{1}H NMR spectrum of **1** in $CDCl_3$.

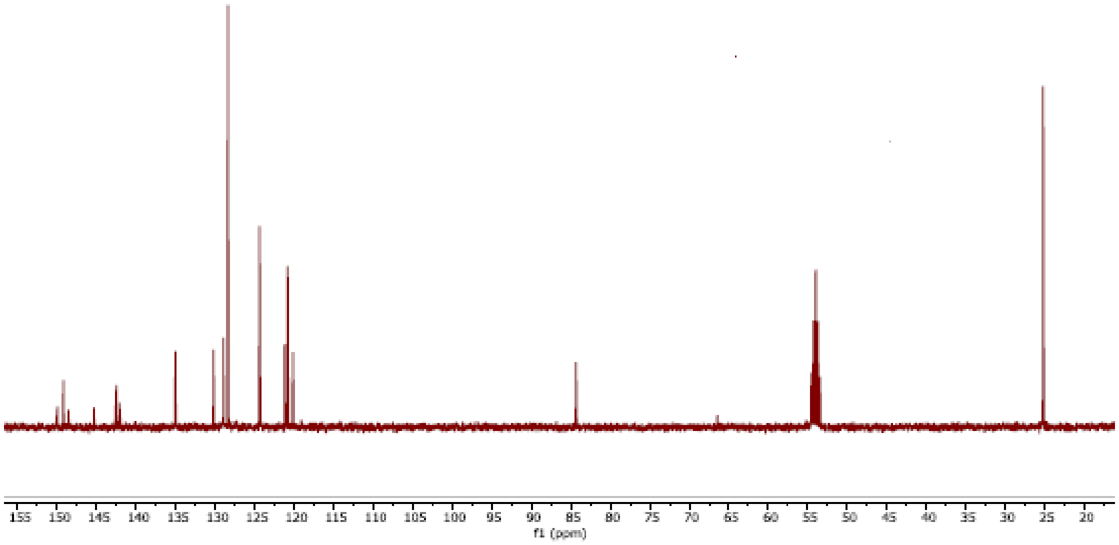

Figure S2. $^{13}C\{^{1}H\}$ NMR spectrum of **1** in $CDCl_3$.

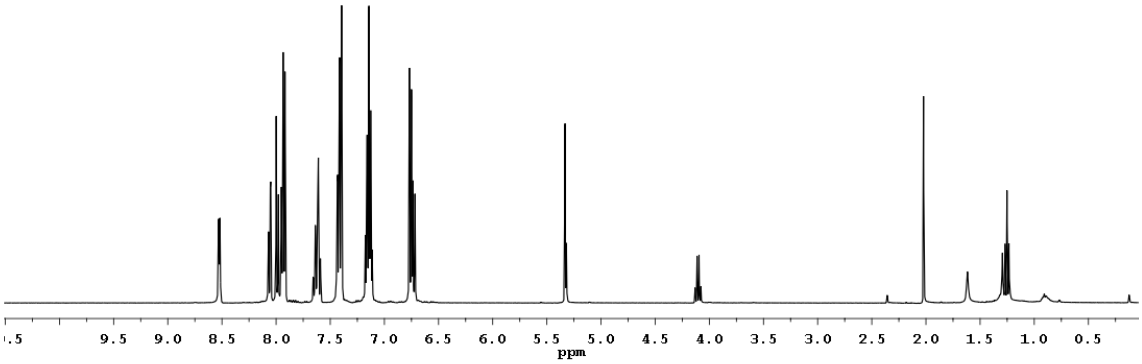

Figure S3. ^{1}H NMR spectrum of **2** in $CDCl_3$.

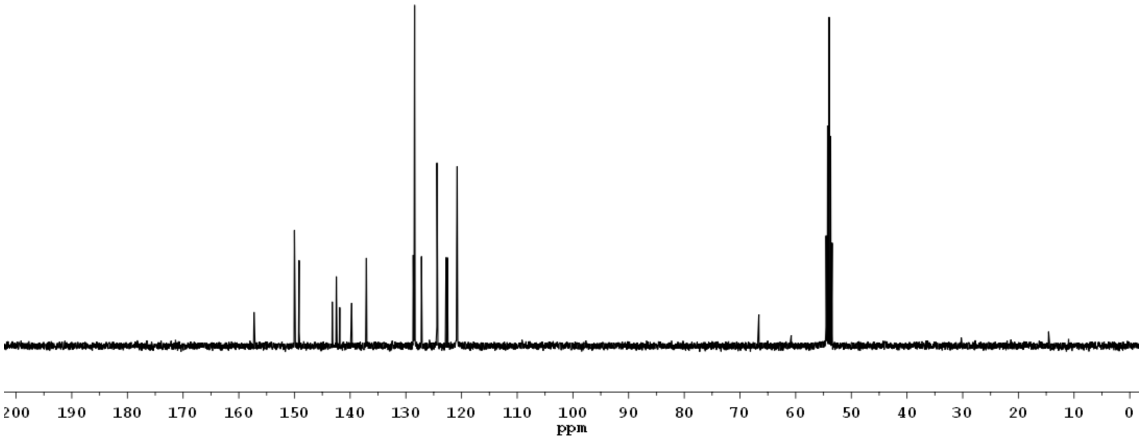

Figure S4. ^{13}C{^{1}H} NMR spectrum of **2** in $CDCl_3$.

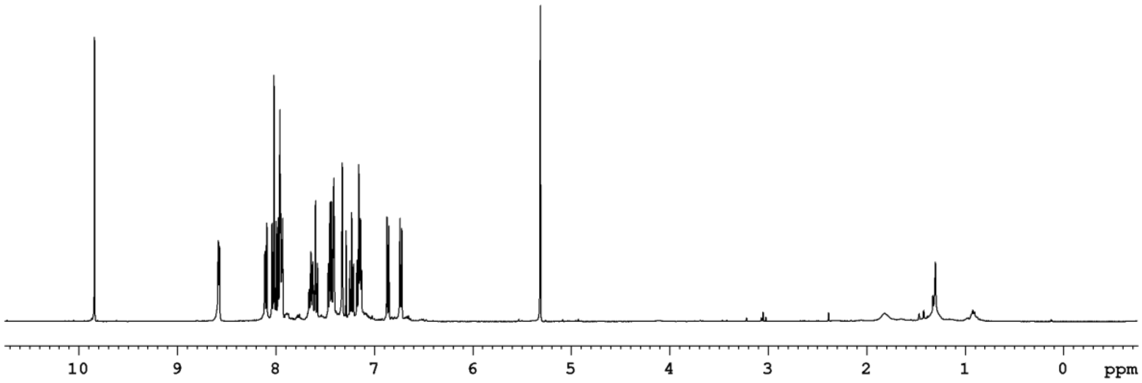

Figure S5. ^{1}H NMR spectrum of **3** in $CDCl_3$.

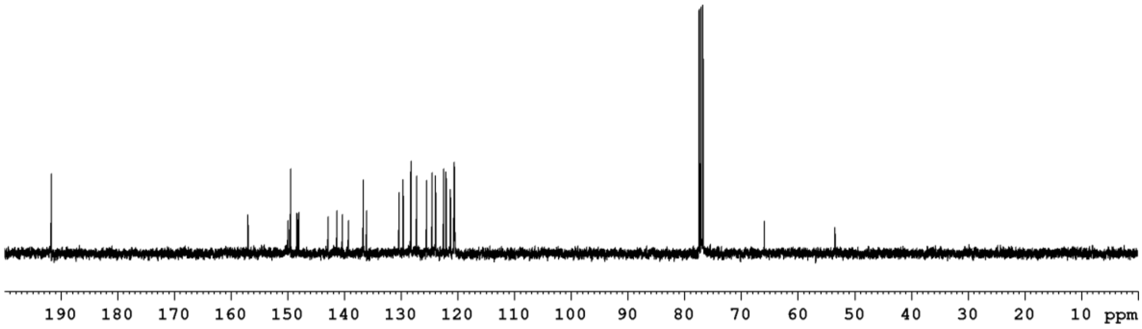

Figure S6. $^{13}C\{^{1}H\}$ NMR spectrum of **3** in $CDCl_3$.

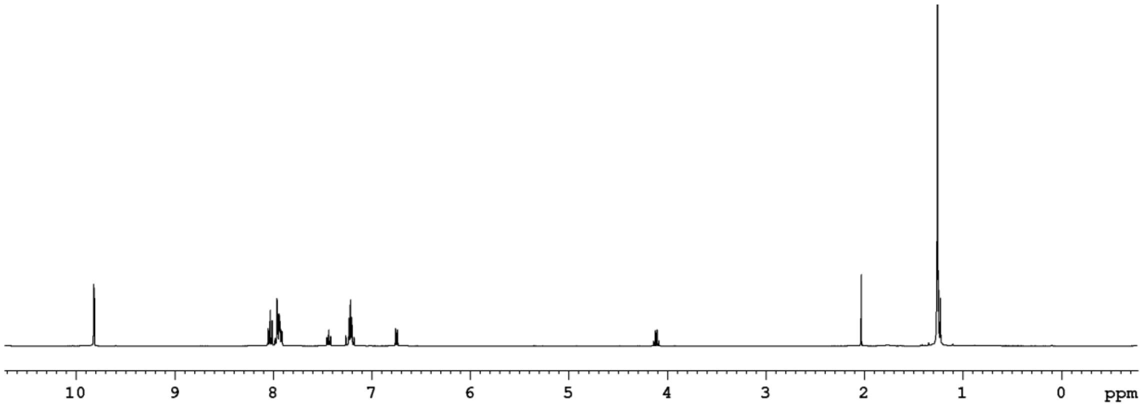

Figure S7. ^{1}H NMR spectrum of **4** in $CDCl_3$.

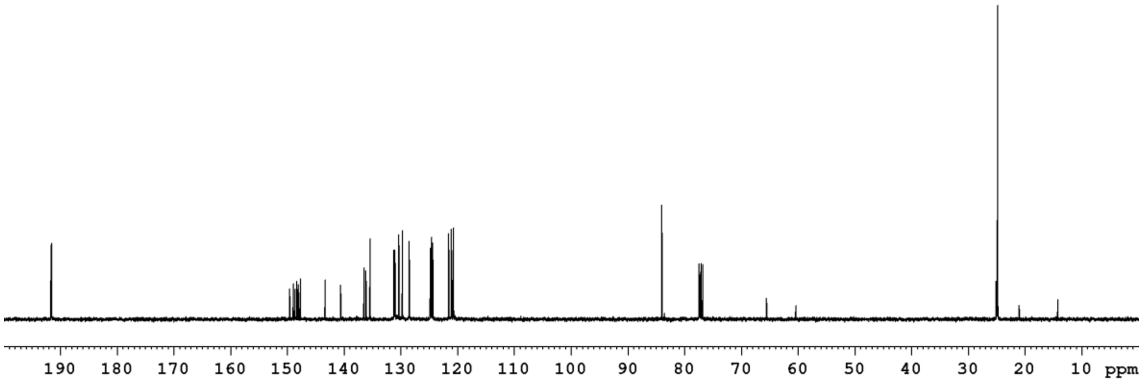

Figure S8. $^{13}C\{^{1}H\}$ NMR spectrum of **4** in $CDCl_3$.

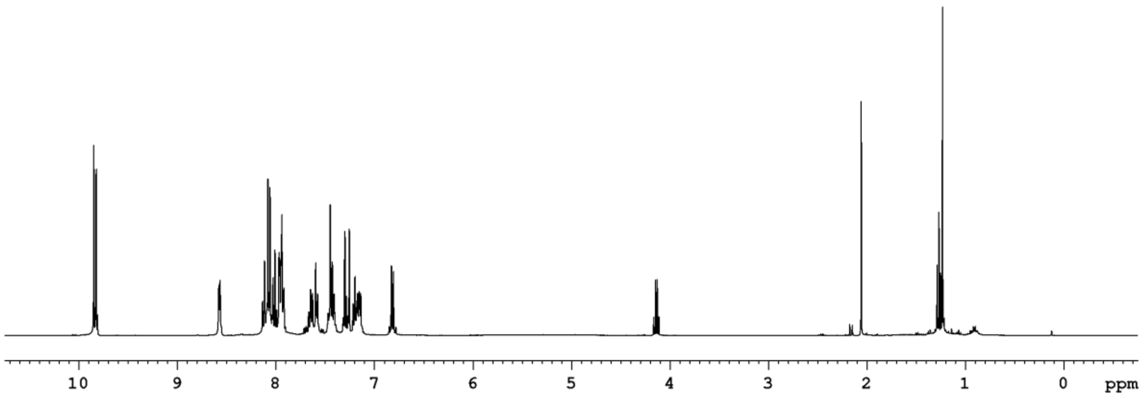

Figure S9. ^{1}H NMR spectrum of **5** in $CDCl_3$.

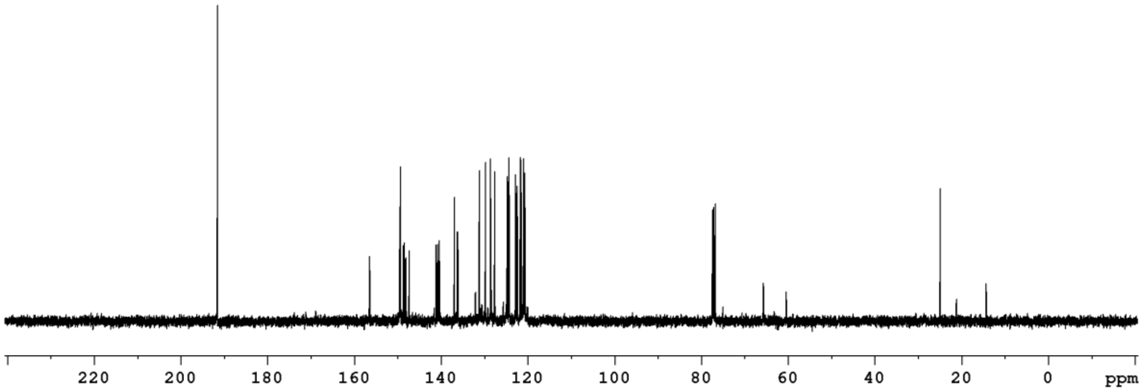

Figure S10. $^{13}C\{^{1}H\}$ NMR spectrum of **5** in $CDCl_3$.

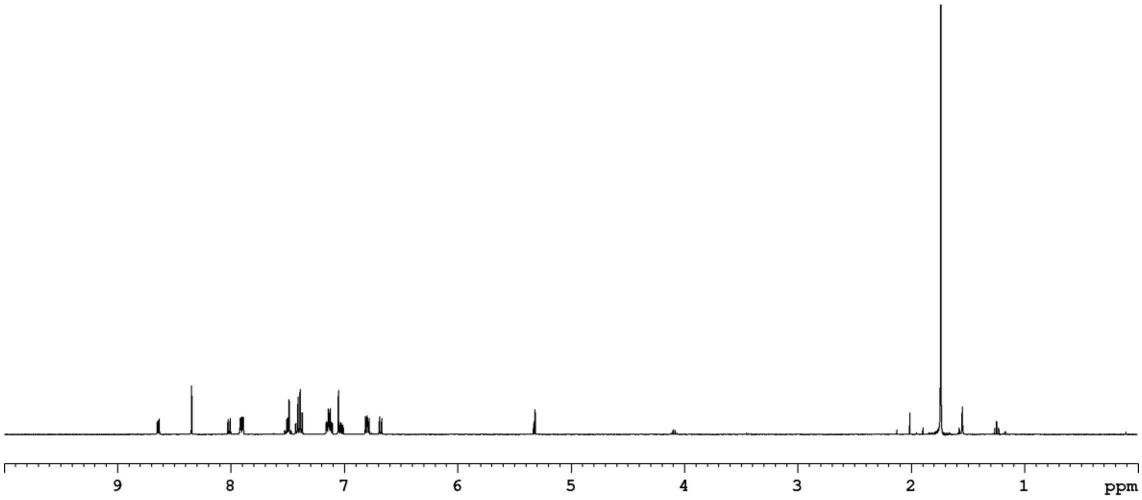

Figure S11. ^{1}H NMR spectrum of the mixture of complexes **Ir6** and **Ir7** in CD_2Cl_2.

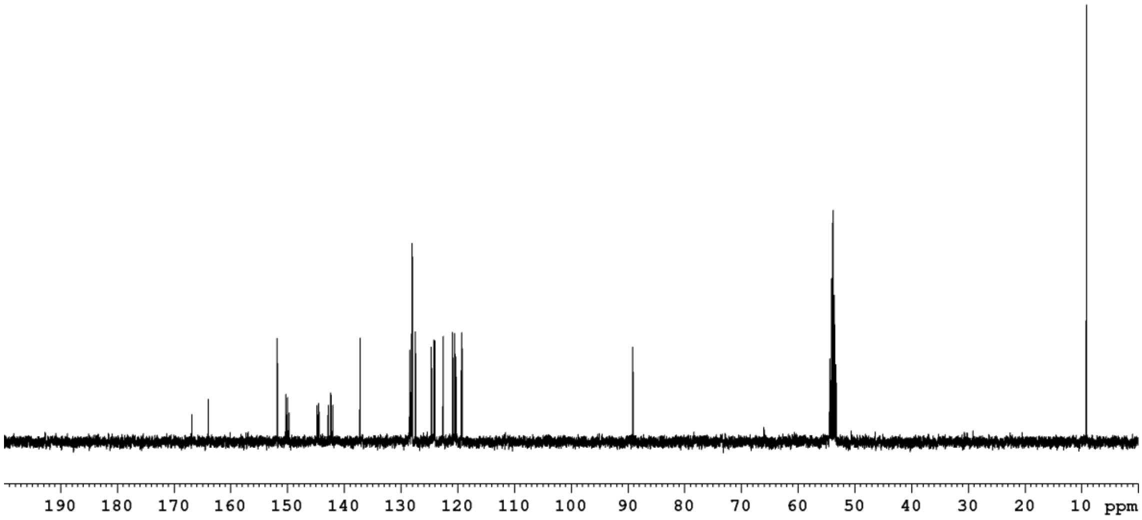

Figure S12. $^{13}C\{^{1}H\}$ NMR spectrum of the mixture of complexes **Ir6** and **Ir7** in CD_2Cl_2.

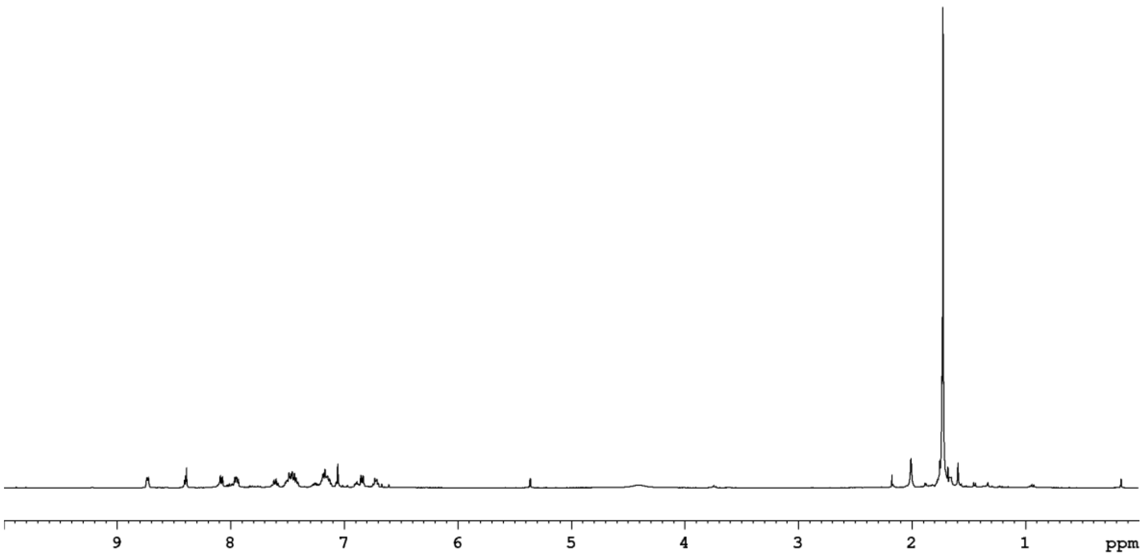

Figure S13. ^{1}H NMR spectrum of the mixture of complexes **Rh6** and **Rh7** in CD_2Cl_2.

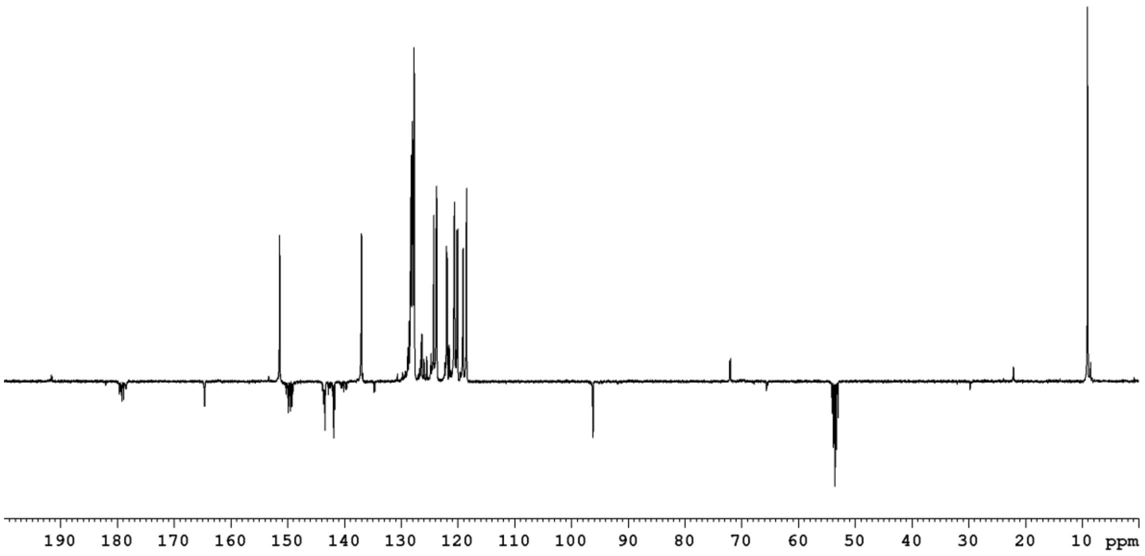

Figure S14. JMOD-$^{13}C\{^1H\}$ NMR spectrum of the mixture of complexes **Rh6** and **Rh7** in CD_2Cl_2.

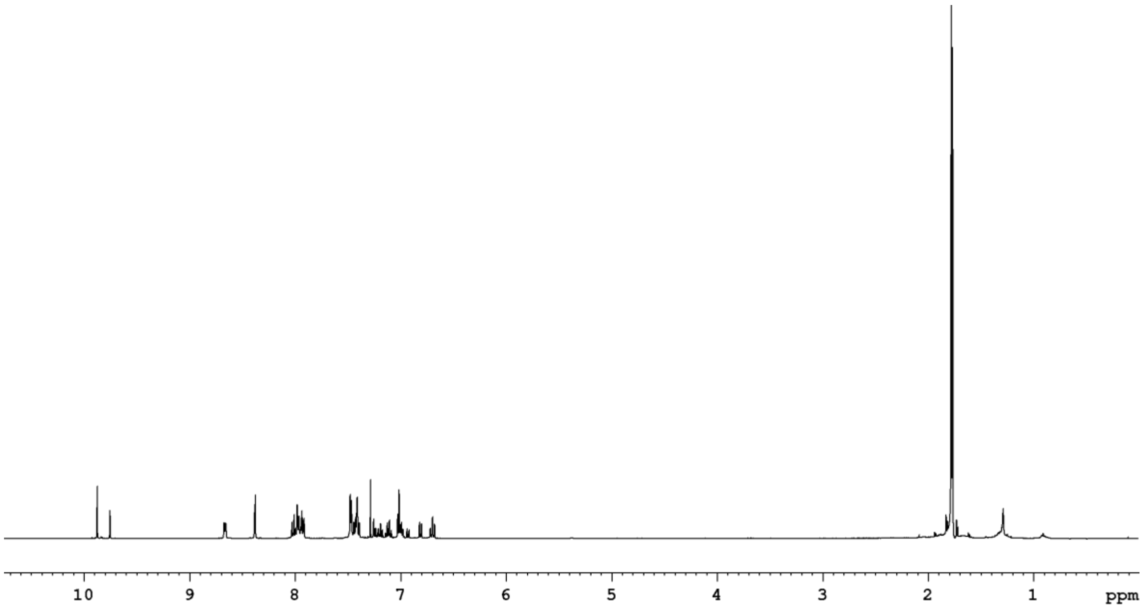

Figure S15. ^{1}H NMR spectrum of the mixture of diastereoisomers (*R*,*M*)* and (*R*,*P*)*-**Ir8** in $CDCl_3$.

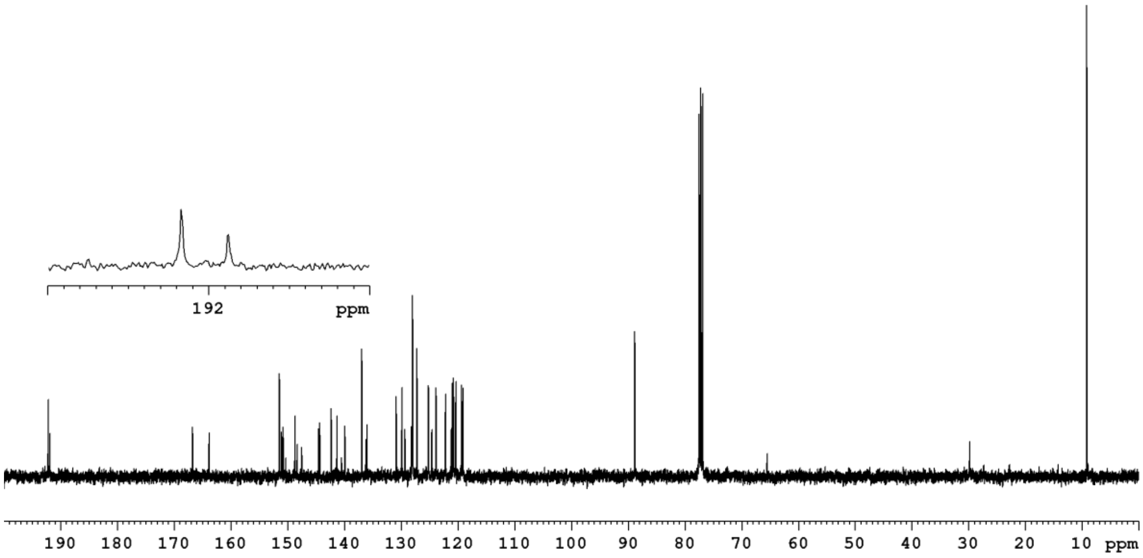

Figure S16. $^{13}C\{^{1}H\}$ NMR spectrum of the mixture of diastereoisomers (*R*,*M*)* and (*R*,*P*)*-**Ir8** in $CDCl_3$.

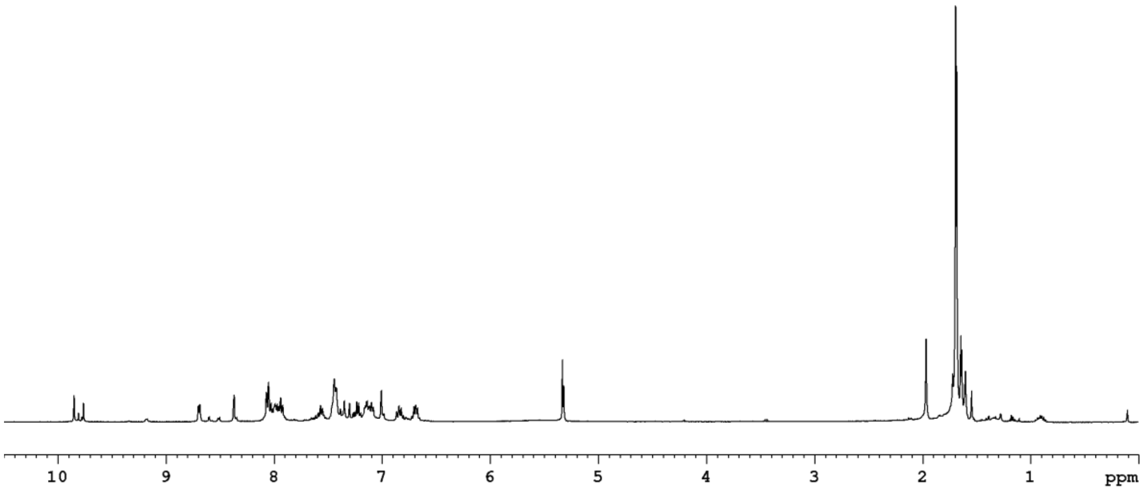

Figure S17. ^{1}H NMR spectrum of the mixture of diastereoisomers (*R*,*M*)* and (*R*,*P*)*-**Rh8** in CD_2Cl_2.

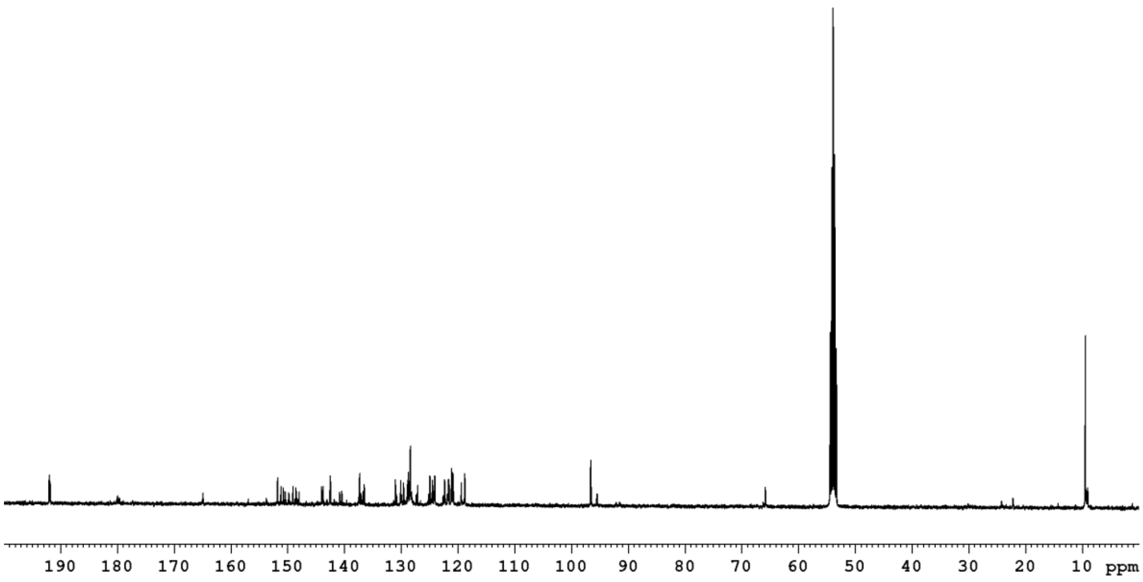

Figure S18. $^{13}C\{^1H\}$ NMR spectrum of the mixture of diastereoisomers (*R*,*M*)* and (*R*,*P*)*-**Rh8** in CD_2Cl_2.

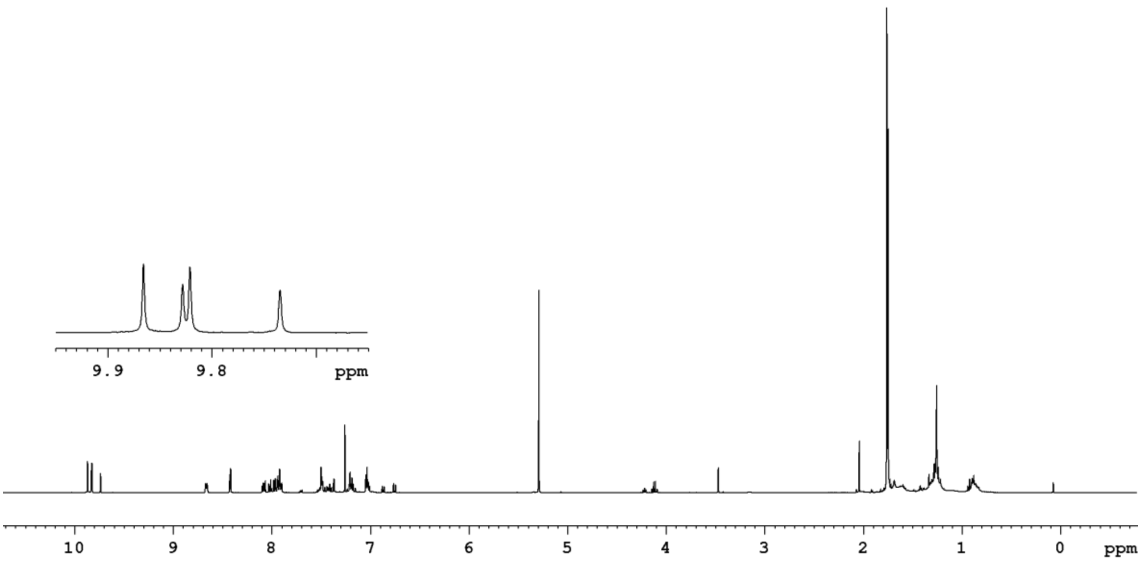

Figure S19. ^{1}H NMR spectrum of the mixture of diastereoisomers (*R*,*M*)* and (*R*,*P*)*-**Ir9** in $CDCl_3$.

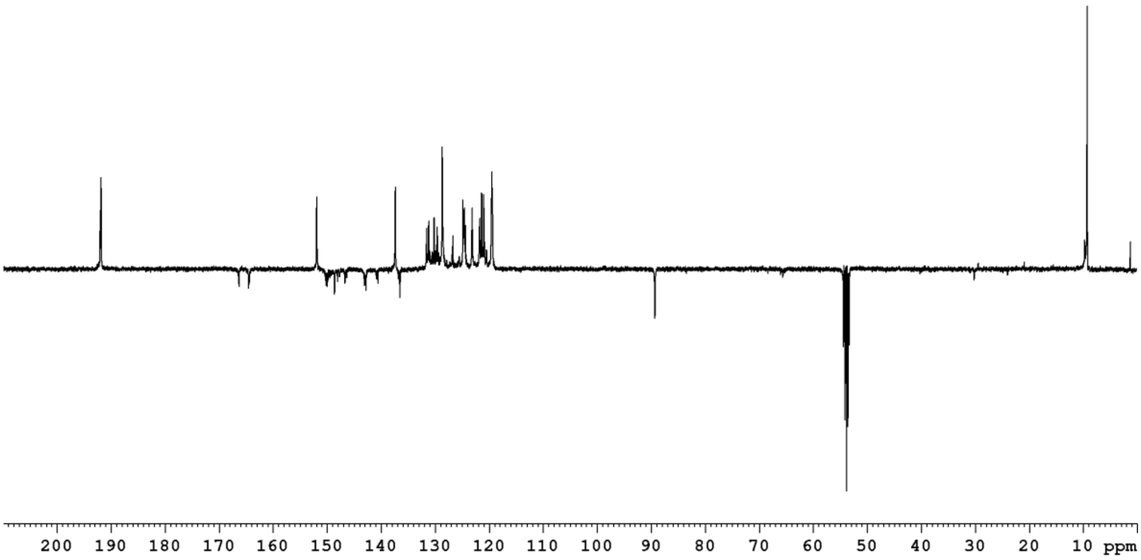

Figure S20. JMOD-$^{13}C\{^{1}H\}$ NMR spectrum of the mixture of diastereoisomers (*R*,*M*)* and (*R*,*P*)*-**Ir9** in $CDCl_3$.

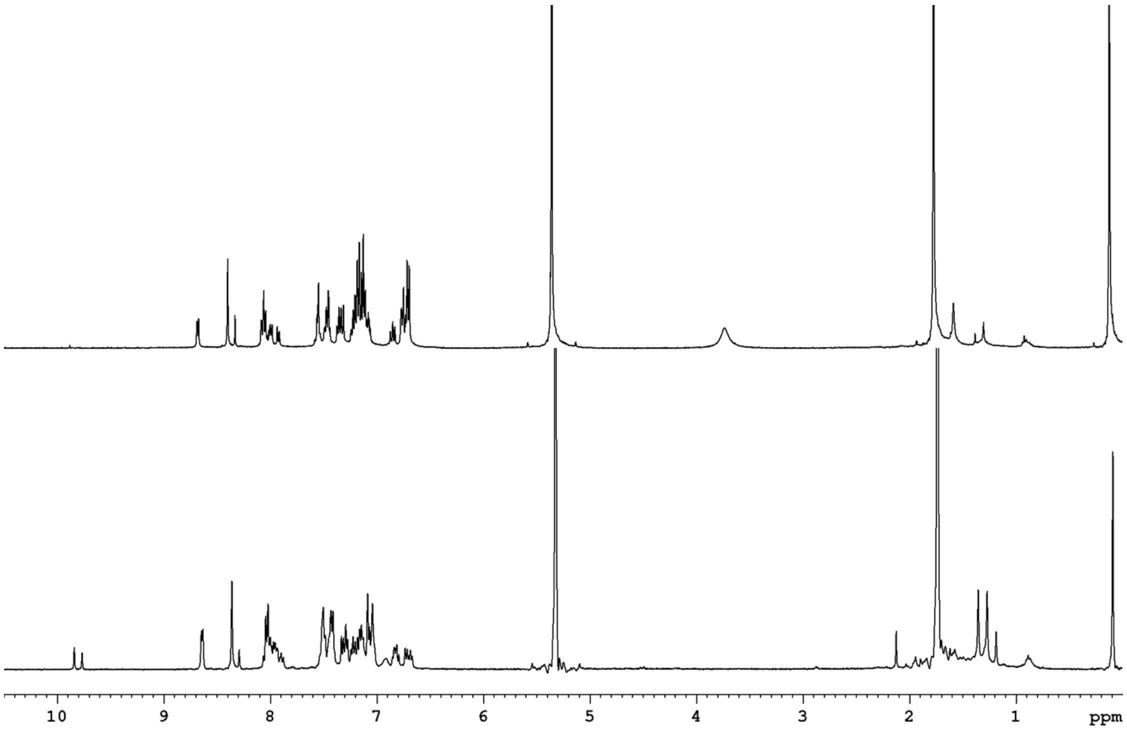

Figure S21. ^{1}H NMR spectrum of the mixture of diastereoisomers (*R*,*M*)* and (*R*,*P*)*-**Ir10** in CD_2Cl_2 in the reaction mixture (top) and after elimination of excess of aniline (bottom).

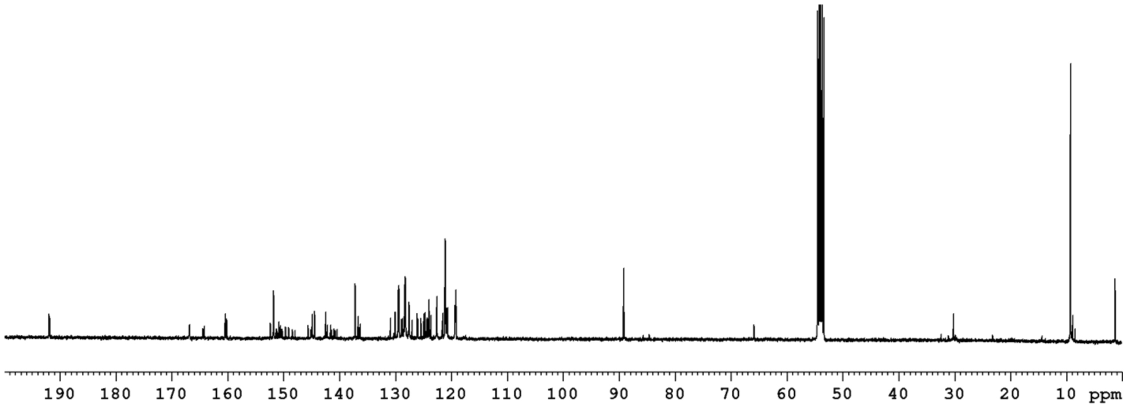

Figure S22. $^{13}C\{^{1}H\}$ NMR spectrum of the mixture of diastereoisomers (*R*,*M*)* and (*R*,*P*)*-**Ir10** in CD_2Cl_2 in the reaction mixture.

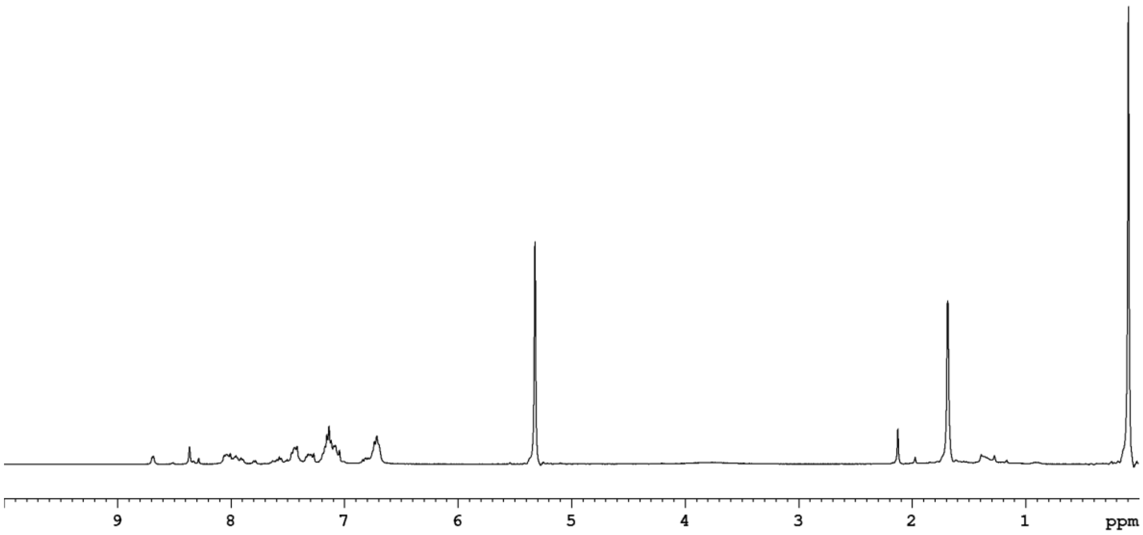

Figure S23. ^{1}H NMR spectrum of the mixture of diastereoisomers (*R*,*M*)* and (*R*,*P*)*-**Rh10** in CD_2Cl_2.

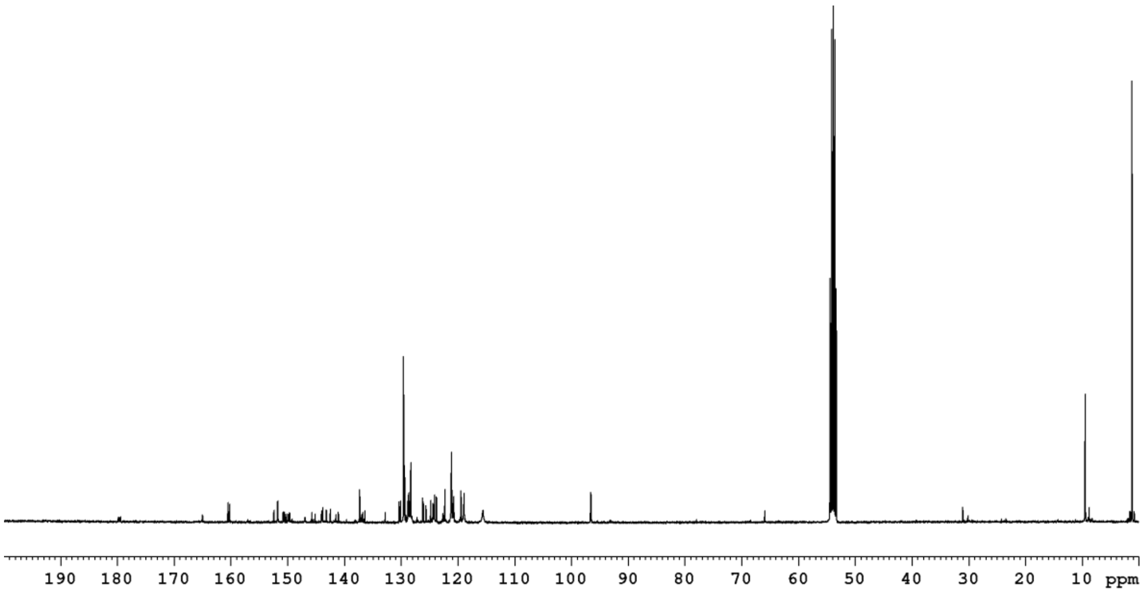

Figure S24. $^{13}C\{^{1}H\}$ NMR spectrum of the mixture of diastereoisomers (*R*,*M*)* and (*R*,*P*)*-**Rh10** in CD_2Cl_2.

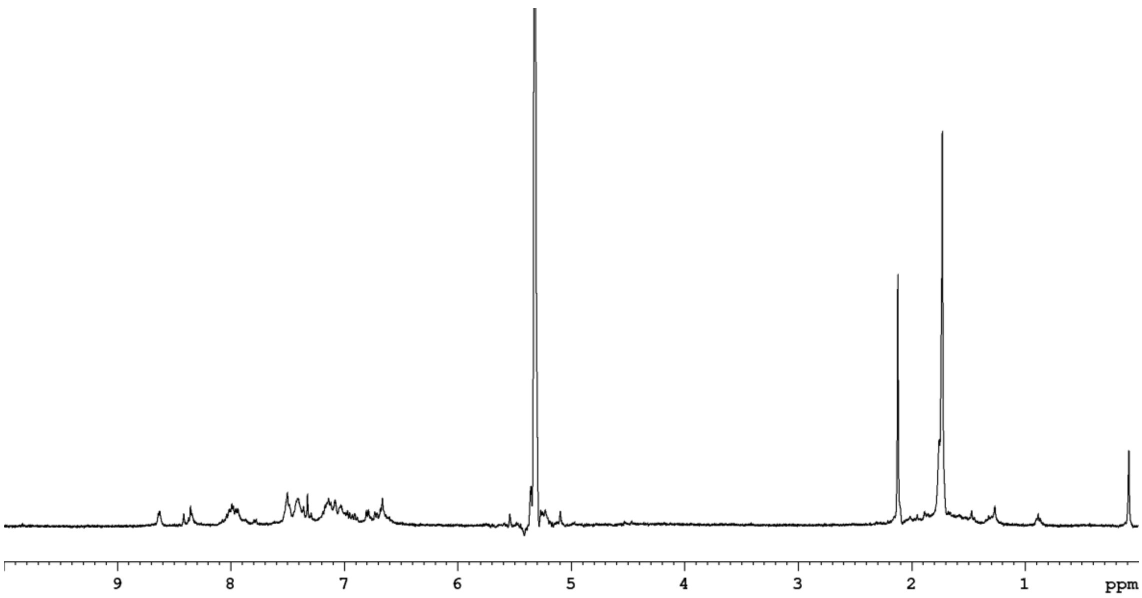

Figure S25. ^{1}H NMR spectrum of the mixture of diastereoisomers (*R*,*M*)* and (*R*,*P*)*-**Ir11** in CD_2Cl_2.

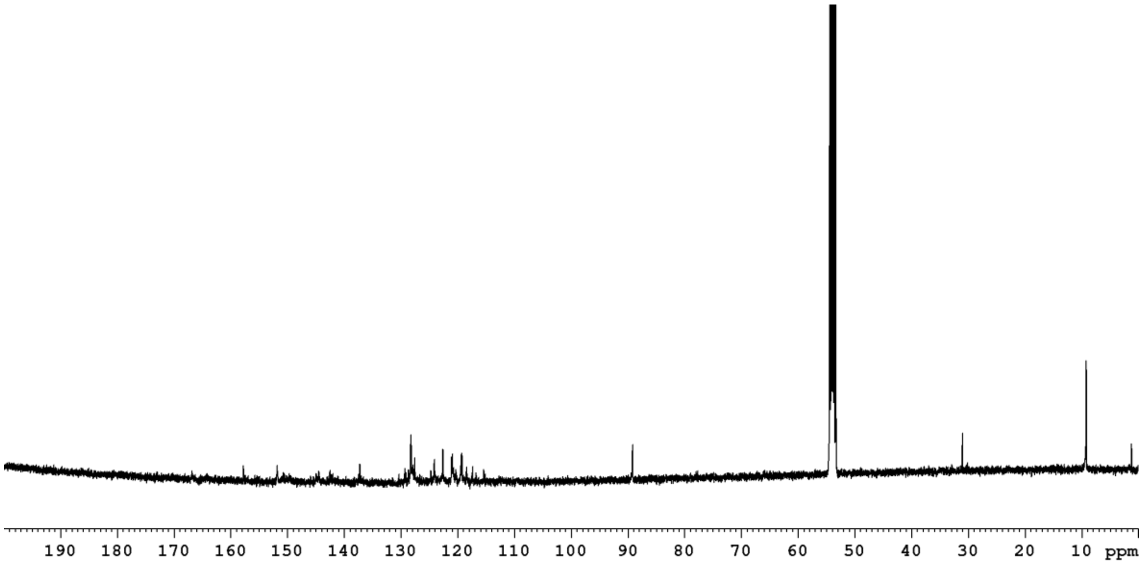

Figure S26. $^{13}C\{^{1}H\}$ NMR spectrum of the mixture of diastereoisomers (*R*,*M*)* and (*R*,*P*)*-**Ir11** in CD_2Cl_2.

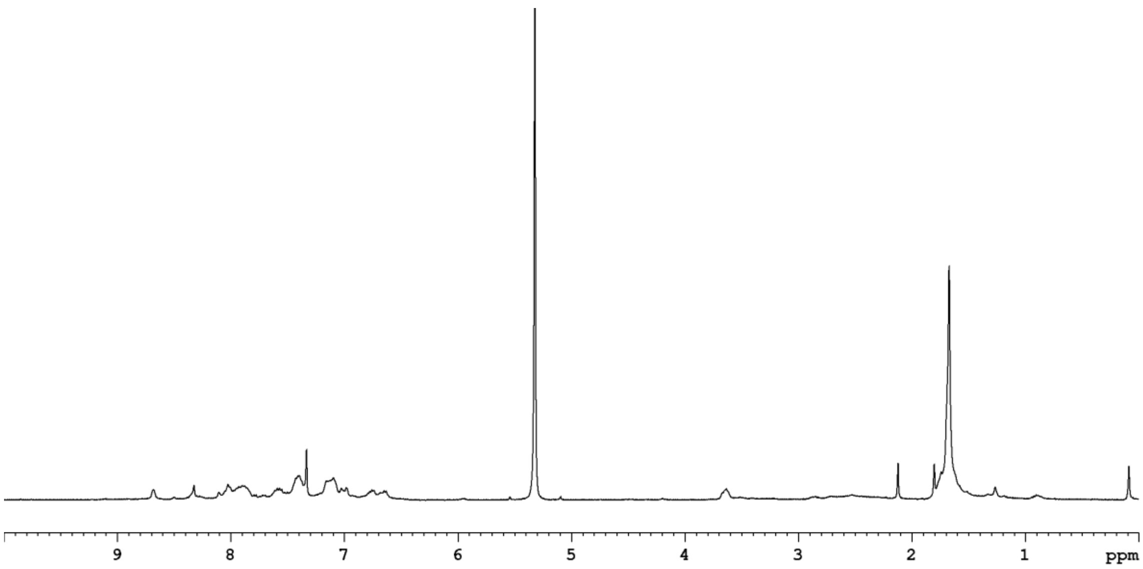

Figure S27. ^{1}H NMR spectrum of the mixture of diastereoisomers (*R*,*M*)* and (*R*,*P*)*-**Rh12** in CD_2Cl_2.

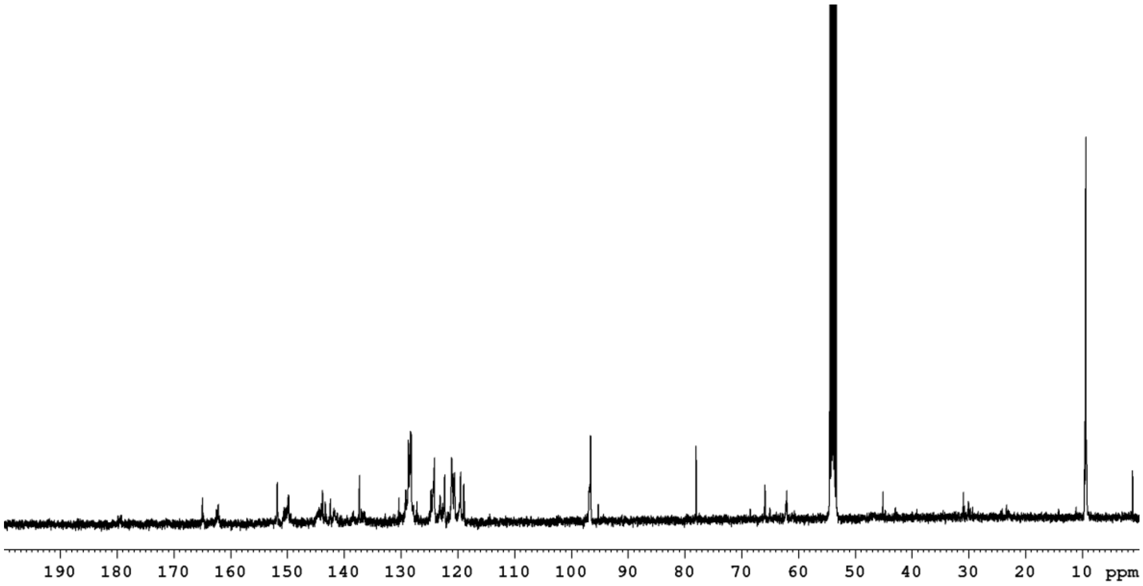

Figure S28. $^{13}C\{^{1}H\}$ NMR spectrum of the mixture of diastereoisomers (*R*,*M*)* and (*R*,*P*)*-**Rh12** in CD_2Cl_2.

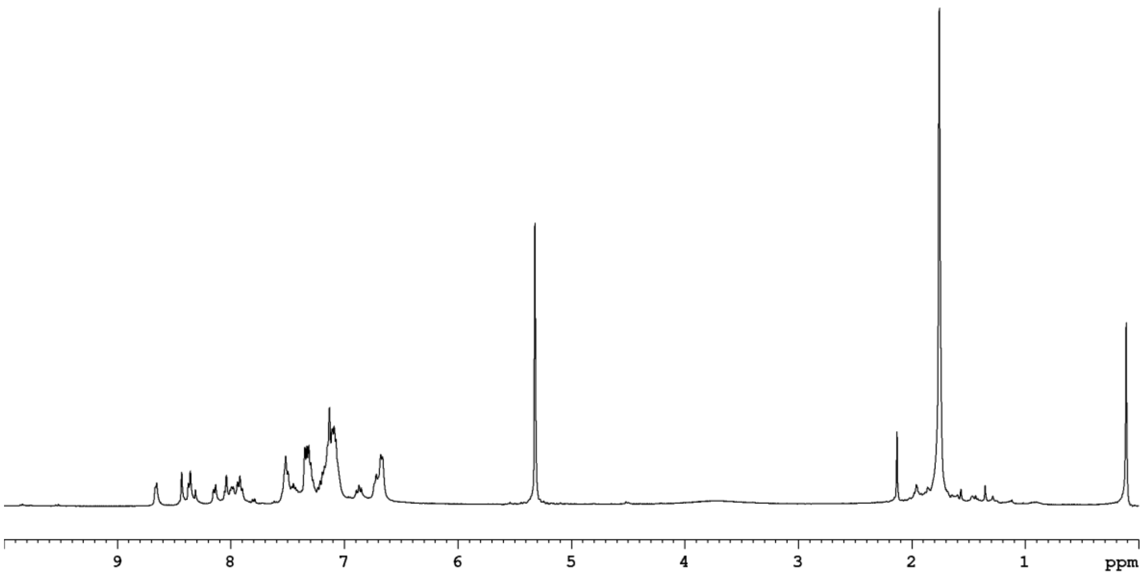

Figure S29. ^{1}H NMR spectrum of the mixture of diastereoisomers (*R*,*M*)* and (*R*,*P*)*-**Ir13** in CD_2Cl_2.

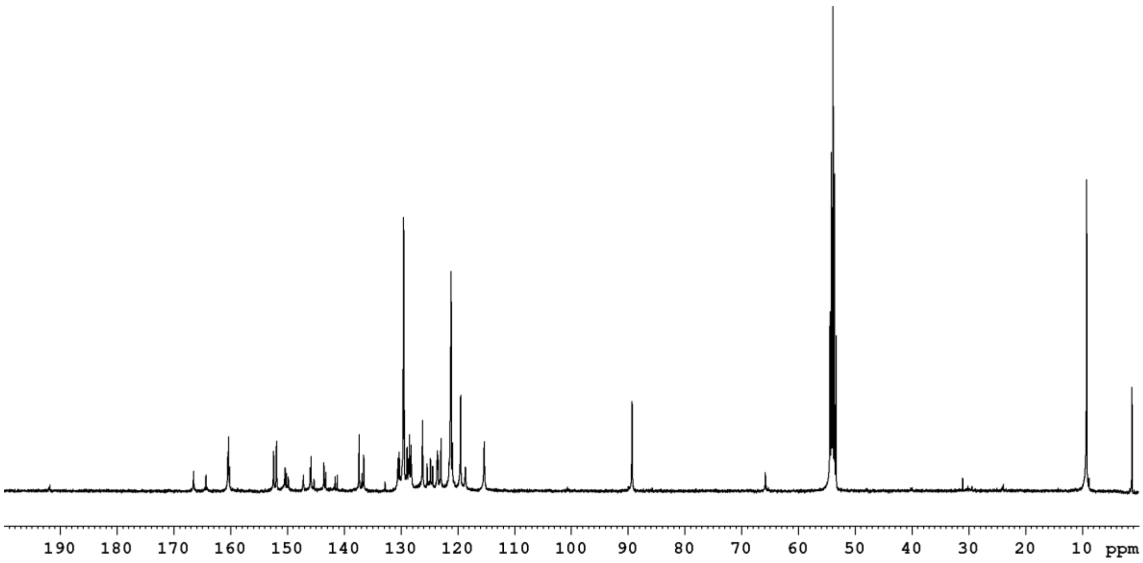

Figure S30. $^{13}C\{^{1}H\}$ NMR spectrum of the mixture of diastereoisomers (*R*,*M*)* and (*R*,*P*)*-**Ir13** in CD_2Cl_2.

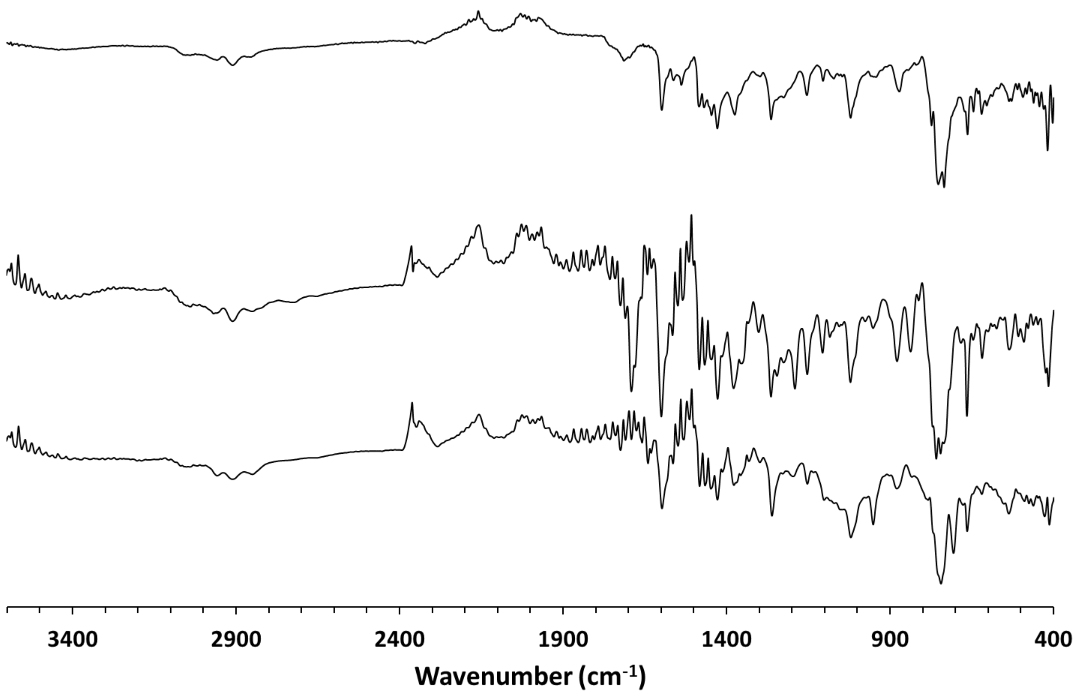

Figure S31. Comparison of IR spectra of compounds **Rh6** + **Rh7** (top), **Rh8** (middle) and **Rh12** (bottom).

Table S1. Crystal data and structure refinement for complexes **Ir6** and **Rh6**.

Complex	**Ir6**	**Rh6**
Empirical formula	$C_{40}H_{33}ClIrN$	$C_{40}H_{33}ClRhN$
Formula weight	755.32	666.03
Temperature	100(2) K	100(2) K
Wavelength	0.71073 Å	0.71073 Å
Crystal system	Monoclinic	Monoclinic
Space group	$P2_1/n$	$P2_1/n$
Unit cell dimensions	a = 12.0032(10) Å	a = 11.9625(6) Å
	b = 10.9034(9) Å	b = 10.9076(6) Å
	c = 23.9599(19) Å	c = 23.9953(13) Å
	α= 90°	α= 90°
	β= 98.608(2)°	β= 98.169(2)
	γ = 90°	γ = 90°
Volume	3100.4(4) $Å^3$	3099.2(3) $Å^3$
Z	4	4
Density (calculated)	1.618 Mg/m3	1.427 Mg/m^3
Absorption coefficient	4.422 mm-1	0.667 mm^{-1}
F(000)	1496	1368
Crystal size	0.126 × 0.119 × 0.085 mm^3	0.217 × 0.132 × 0.0252 mm^3
θ range for data collection	2.537 to 28.357°	2.535 to 28.371°
Index ranges	-16<=h<=13, -14<=k<=14, -231<=l<=32	-15<=h<=15, -14<=k<=14, -30<=l<=32
Reflections collected	48021	49113
Independent reflections	7727 [R(int) = 0.0529]	7732 [R(int) = 0.0565]
Completeness to θ = 25.242°	99.9 %	99.9 %
Refinement method	Full-matrix least-squares on F^2	
Data / restraints / parameters	7727 / 0 / 393	7732 / 0 / 393
Goodness-of-fit on F^2	1.106	1.044
Final R indices [I>2σ(I)]	R_1 = 0.0304, wR_2 = 0.0569	R_1 = 0.0376, wR_2 = 0.0875
R indices (all data)	R_1 = 0.0403, wR_2 = 0.0590	R_1 = 0.0534, wR_2 = 0.0929
Largest diff. peak and hole	1.694 and -1.978 e.$Å^{-3}$	2.003 and -0.598 e.$Å^{-3}$
